# MurA escape mutations uncouple peptidoglycan biosynthesis from PrkA signaling

**DOI:** 10.1371/journal.ppat.1010406

**Published:** 2022-03-16

**Authors:** Sabrina Wamp, Patricia Rothe, Daniel Stern, Gudrun Holland, Janina Döhling, Sven Halbedel

**Affiliations:** 1 FG11 - Division of Enteropathogenic bacteria and *Legionella*, Robert Koch Institute, Wernigerode, Germany; 2 ZBS3 - Biological Toxins, Robert Koch Institute, Berlin, Germany; 3 ZBS4 - Advanced Light and Electron Microscopy, Robert Koch Institute, Berlin, Germany; University of Massachusetts Medical School, UNITED STATES

## Abstract

Gram-positive bacteria are protected by a thick mesh of peptidoglycan (PG) completely engulfing their cells. This PG network is the main component of the bacterial cell wall, it provides rigidity and acts as foundation for the attachment of other surface molecules. Biosynthesis of PG consumes a high amount of cellular resources and therefore requires careful adjustments to environmental conditions. An important switch in the control of PG biosynthesis of *Listeria monocytogenes*, a Gram-positive pathogen with a high infection fatality rate, is the serine/threonine protein kinase PrkA. A key substrate of this kinase is the small cytosolic protein ReoM. We have shown previously that ReoM phosphorylation regulates PG formation through control of MurA stability. MurA catalyzes the first step in PG biosynthesis and the current model suggests that phosphorylated ReoM prevents MurA degradation by the ClpCP protease. In contrast, conditions leading to ReoM dephosphorylation stimulate MurA degradation. How ReoM controls degradation of MurA and potential other substrates is not understood. Also, the individual contribution of the ~20 other known PrkA targets to PG biosynthesis regulation is unknown. We here present *murA* mutants which escape proteolytic degradation. The release of MurA from ClpCP-dependent proteolysis was able to activate PG biosynthesis and further enhanced the intrinsic cephalosporin resistance of *L*. *monocytogenes*. This latter effect required the RodA3/PBP B3 transglycosylase/transpeptidase pair. One *murA* escape mutation not only fully rescued an otherwise non-viable *prkA* mutant during growth in batch culture and inside macrophages but also overcompensated cephalosporin hypersensitivity. Our data collectively indicate that the main purpose of PrkA-mediated signaling in *L*. *monocytogenes* is control of MurA stability during standard laboratory growth conditions and intracellular growth in macrophages. These findings have important implications for the understanding of PG biosynthesis regulation and β-lactam resistance of *L*. *monocytogenes* and related Gram-positive bacteria.

## Author summary

Peptidoglycan (PG) is the main component of the bacterial cell wall and many of the PG synthesizing enzymes are antibiotic targets. We previously have discovered a new signaling route controlling PG production in the human pathogen *Listeria monocytogenes*. This route also determines the intrinsic resistance of *L*. *monocytogenes* against cephalosporins, a group of β-lactam antibiotics. Signaling involves PrkA, a membrane-embedded protein kinase, that is activated during cell wall stress to phosphorylate its target ReoM. Depending on its phosphorylation, ReoM activates or inactivates PG production by controlling the proteolytic stability of MurA, which catalyzes the first step in PG biosynthesis. MurA degradation depends on the ClpCP protease and we here have isolated *murA* mutations that escape this degradation. Using these mutants, we could show that regulation of PG biosynthesis through control of MurA stability is an important purpose of PrkA-mediated signaling in *L*. *monocytogenes*. Further experiments identified the transglycosylase RodA and the transpeptidase PBP B3 as additional downstream factors. Our results suggest that both proteins act together to translate the signals received by PrkA into adjustment of PG biosynthesis. These findings shed new light on the regulation of PG biosynthesis in Gram-positive bacteria with intrinsic β-lactam resistance.

## Introduction

The cell wall of Gram-positive bacteria is built up from peptidoglycan (PG) strands that are crosslinked with each other to form a three-dimensional network called the sacculus. The sacculus engulfs the entire cell, determines cell shape and provides the key matrix for the attachment of other surface molecules such as teichoic acids, proteins and polymeric carbohydrates. PG biosynthesis is a target of many commonly used antibiotics that can lead to lysis of bacterial cells by weakening the integrity of the sacculus that then gives way to the high internal turgor pressure [1–3]. PG accounts for approximately 20% of the weight of a Gram-positive cell [4], illustrating the high demand PG biosynthesis imposes on biosynthetic and energy supplying pathways. Thus, bacteria need to tightly adjust PG production to changing environmental and growth conditions to prevent unnecessary losses of building blocks and energy.

The first step in PG biosynthesis is mediated by MurA, which initiates a series of eight cytoplasmic reactions that sequentially convert UDP-*N*-acetyl-glucosamine (UDP-Glc*N*Ac) into a lipid-linked disaccharide carrying a pentapeptide side chain (lipid II). Lipid II is flipped to the other side of the membrane by Amj- or MurJ-like flippases [5–7], where the disaccharide unit is incorporated into the growing PG chain and the pentapeptides crosslinked either through bifunctional penicillin binding proteins (PBPs) providing transglycosylase and transpeptidase activity [8,9] or through FtsW/RodA-like transglycosylases that act in concert with monofunctional PBPs, which are mere transpeptidases [10–13].

Among the various regulatory mechanisms controlling PG production, PASTA (PBP and serine/threonine kinase associated) domain-containing eukaryotic-like serine/threonine protein kinases (PASTA-eSTKs) have an outstanding role in PG biosynthesis regulation of firmicutes and actinobacteria, two major types of Gram-positives [14–16]. PASTA-eSTKs sense cell wall damaging conditions by interaction of lipid II with their extracellular PASTA domains, and this leads to the activation of their cytosolic kinase domain [17,18]. In actinobacteria, many of the kinase substrates directly participate in PG biosynthesis, such as GlmU, important for biosynthesis of UDP-Glc*N*Ac, the flippase MurJ (MviN) or the bifunctional PBP PonA1 [19–21]. Furthermore, actinobacteria control MurA activity by PASTA-eSTK-dependent phosphorylation of CwlM, which allosterically activates MurA [22,23].

Interestingly, MurA is also the target of PASTA-eSTK-dependent regulation in firmicutes. This involves ReoM, which we have recently described as a novel substrate of the PASTA-eSTK PrkA in *Listeria monocytogenes* [24], a foodborne pathogen causing life-threatening infections [25]. We originally identified *reoM* in a screen for suppressors of the heat-sensitive phenotype of a *L*. *monocytogenes* mutant lacking the late cell division protein GpsB [24,26,27]. An *L*. *monocytogenes* Δ*reoM* mutant has increased ceftriaxone resistance and thicker polar PG layers indicating activated PG biosynthesis [24]. PrkA phosphorylates ReoM *in vitro* at the conserved Thr-7 residue and the phosphatase PrpC reverses this [24]. PrkA-dependency of the ReoM phosphorylation was also demonstrated *in vivo* [28]. ReoM is conserved in firmicutes but absent from actinobacteria [24,29]. It does not act as an allosteric activator of MurA but rather controls proteolytic stability of MurA together with ReoY, a second new factor that was identified in the same screen [24]. In *L*. *monocytogenes* and *Bacillus subtilis*, MurA is a substrate of the ClpCP protease [30,31] and *reoM* and *reoY* mutants accumulate MurA to a similar extent as a *clpC* mutant [24,27]. Analysis of phospho-ablative *reoM* strains and mutants depleted for PrkA and the corresponding protein phosphatase PrpC showed that genetic constellations, in which ReoM is locked in the phosphorylated state, cause MurA stabilization and *vice versa* [24]. Thus, PrkA activation leads to activation of PG biosynthesis in firmicutes and actinobacteria even though through different mechanisms.

ReoM interacts with MurA and ReoY in a bacterial two hybrid system and ReoY in turn binds ClpC and ClpP [24]. We hypothesized that ReoM and ReoY have an adaptor-like function and present MurA for degradation to the ClpCP protease, but how these proteins exactly contribute to MurA degradation is not known.

We here describe the identification of novel *gpsB* suppressors. The subsequent in-depth analysis of these suppressor mutants led to the identification of MurA variants that escape proteolytic degradation. We use these mutants to demonstrate that control of MurA degradation by ReoM and ClpCP is the major regulatory pathway of PrkA-dependent signaling in *L*. *monocytogenes* in batch culture as well as during macrophage infection.

## Results

### Novel *gpsB* suppressor mutations in *murA* and *prpC*

**Fig 1:**
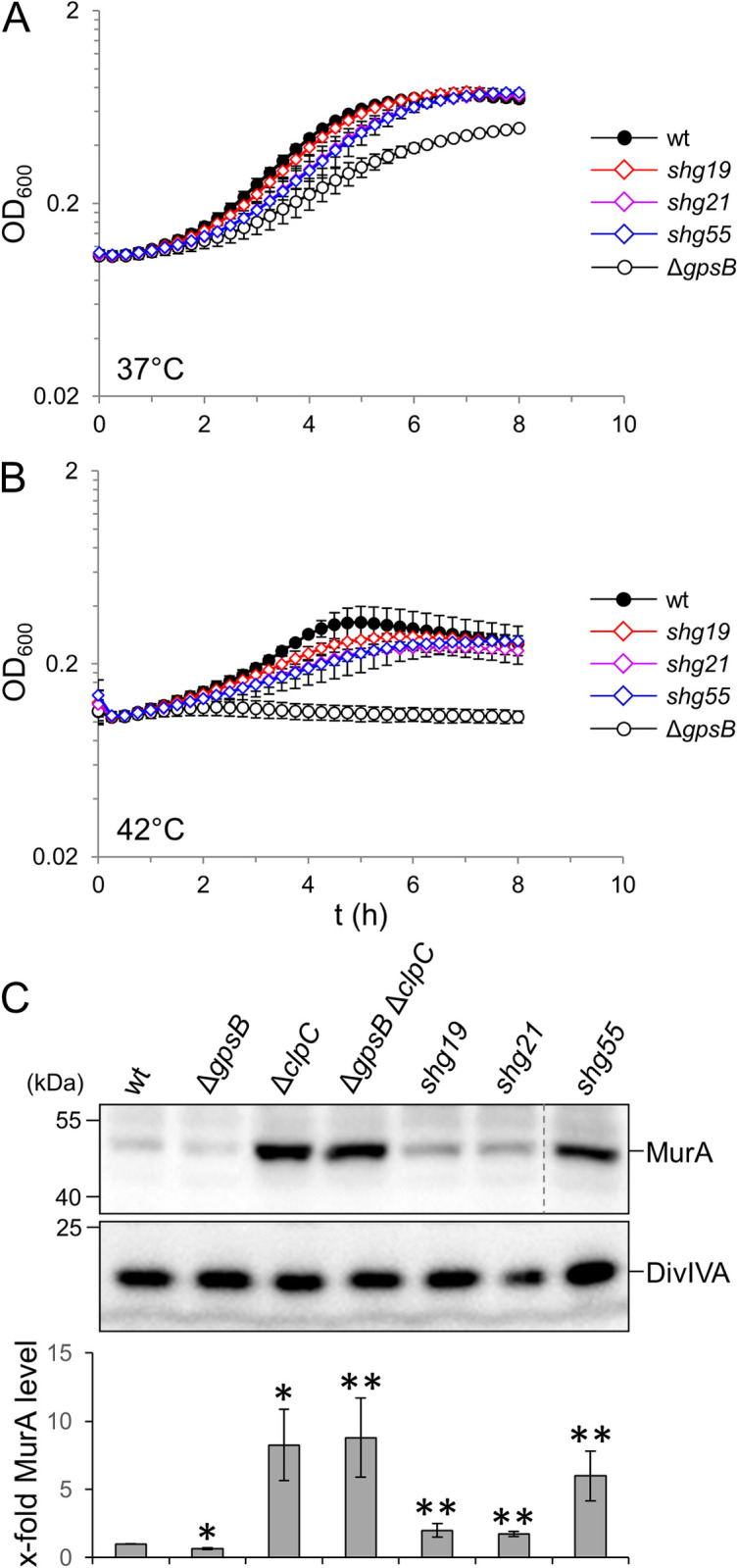
Suppression of the Δ*gpsB* growth defect by *murA* and *prpC* mutations. (A-B) Growth of *L*. *monocytogenes* strains with mutations in *gpsB*, *murA* and *prpC*. *L*. *monocytogenes* strains EGD-e (wt), LMJR19 (Δ*gpsB*), *shg19* (Δ*gpsB murA S262L*), *shg21* (Δ*gpsB murA N197D*) and *shg55* (Δ*gpsB prpC*^*1-44*^) were grown in BHI broth at 37°C (A) and 42°C (B). The experiment was performed in triplicate and average values and standard deviations are shown. (C) Western blots showing MurA and DivIVA levels (for control) in the same set of strains. Strains LMJR138 (Δ*clpC*) and LMJR139 (Δ*gpsB* Δ*clpC*) were included as controls. Equal amounts of total cellular proteins were loaded. Irrelevant lanes were removed (dashed line). MurA signals from three independent experiments were quantified by densitometry and average values and standard deviations are shown. Asterisks indicated statistically significant differences compared to wild type (*) or compared to the Δ*gpsB* mutant (**, *P*<0.05, *t*-test with Bonferroni-Holm correction).

An *L*. *monocytogenes* Δ*gpsB* mutant has a temperature sensitive growth phenotype and cannot multiply at 42°C [26]. However, this mutant readily picks up *shg* suppressor mutations (suppression of heat-sensitive growth) correcting this growth defect and known *shg* suppressor mutations map to *clpC*, *murZ*, *reoM* and *reoY*, all involved in regulation of MurA stability by ClpCP-dependent proteolysis [24,27]. In order to identify further mutations suppressing the *gpsB* phenotype, the *L*. *monocytogenes* Δ*gpsB* mutant strain LMJR19 was streaked on BHI plates and incubated at 42°C. After two days of incubation, 50 suppressors were isolated and streaked to single colonies. The *clpC*, *murZ*, *reoM* and *reoY* genes of these 50 suppressors were amplified by PCR and sequenced by Sanger sequencing. Seven suppressors had wild type alleles of the four known *gpsB* suppressor genes and thus must have acquired novel *shg* suppressor mutations somewhere else on the chromosome. Genome sequencing of these seven *shg* suppressors revealed unique mutations mapping either to *murA* or to *prpC*. While the *murA* gene was affected by mutations introducing amino acid substitutions only (*shg19*: *murA S262L*, *shg21*: *murA N197D*), the *shg* mutations in *prpC* introduced amino acid exchanges (*shg24*: *prpC N83S L125F*, *shg47*: *prpC G39S*) as well as premature stop codons (*shg32*: *prpC*^*1-109*^, *shg42*: *prpC*^*1-73*^, *shg55*: *prpC*^*1-44*^). This observation is in good agreement with the reported essentiality of *murA* [27], but conflicts with our previous observation that *prpC* could not be removed from the *L*. *monocytogenes* genome [24]. Suppressor strains *shg19* (Δ*gpsB murA S262L*), *shg21* (Δ*gpsB murA N197D*) and *shg55* (Δ*gpsB prpC*^*1-44*^) were chosen for further experiments and growth was recorded at 37°C and 42°C. All three suppressor strains grew almost like wild type at 37°C and 42°C, whereas the Δ*gpsB* mutant grew with delayed rate at 37°C and did not grow at 42°C (Fig 1A and 1B).

Suppression in *shg* suppressors can involve proteolytic stabilization of MurA leading to stimulation of PG biosynthesis. In order to test, whether MurA is stabilized in the novel *shg* suppressors, their cellular MurA levels were determined by Western blotting. This revealed a mild increase of the MurA level in *shg19* (2.1±0.6-fold compared to wild type) and *shg21* (1.8±0.1-fold) and a strong increase in *shg55* (6.6±1.7-fold), which is almost as strong as observed in a Δ*clpC* mutant (9.1±2.7-fold, Fig 1C). Taken together, we have identified novel *shg* suppressor mutations in *murA* and *prpC* affecting the cellular levels of MurA.

### Deletion of *prpC* is tolerated in the Δ*gpsB* background

Previous results demonstrated that *prpC* could not be removed from the chromosome unless compensatory *prkA* mutations reduced the activity of the cognate protein kinase [24]. As this suggested that *prpC* represents an essential gene in *L*. *monocytogenes*, we were surprised to see that *prpC* repeatedly was inactivated by the introduction of premature stop codons in the *gpsB* suppressor mutants *shg32*, *shg42* and *shg55*. To test whether *prpC* becomes dispensable in the absence of *gpsB*, we tried to delete *prpC* in the background of the Δ*gpsB* mutant using integration/re-excision of a temperature sensitive plasmid designed to remove the *prpC* gene during excision from the chromosome. Plasmid excision is supposed to generate a 50:50 mixture of wild type and deletion mutant clones [32]. Unlike in previous *prpC* deletion attempts in wild type background [24], *prpC* could be readily deleted in the Δ*gpsB* mutant. Furthermore, it even seemed that *prpC* deletion is favored in the absence of *gpsB*, as 19 out of 20 tested clones had factually lost *prpC*. Unlike the Δ*gpsB* single mutant, the resulting Δ*gpsB* Δ*prpC* double mutant could grow at 42°C, even though it did not fully reach normal wild type growth (S1A Fig). Moreover, MurA levels increased 2.8±0.5-fold in the Δ*gpsB* Δ*prpC* double mutant compared to wild type (S1B Fig). These data confirm that inactivation of *prpC* suppresses the Δ*gpsB* phenotype and that suppression is likely explained by accumulation of MurA.

### Effect of N197D and S262L mutations on MurA activity

**Fig 2:**
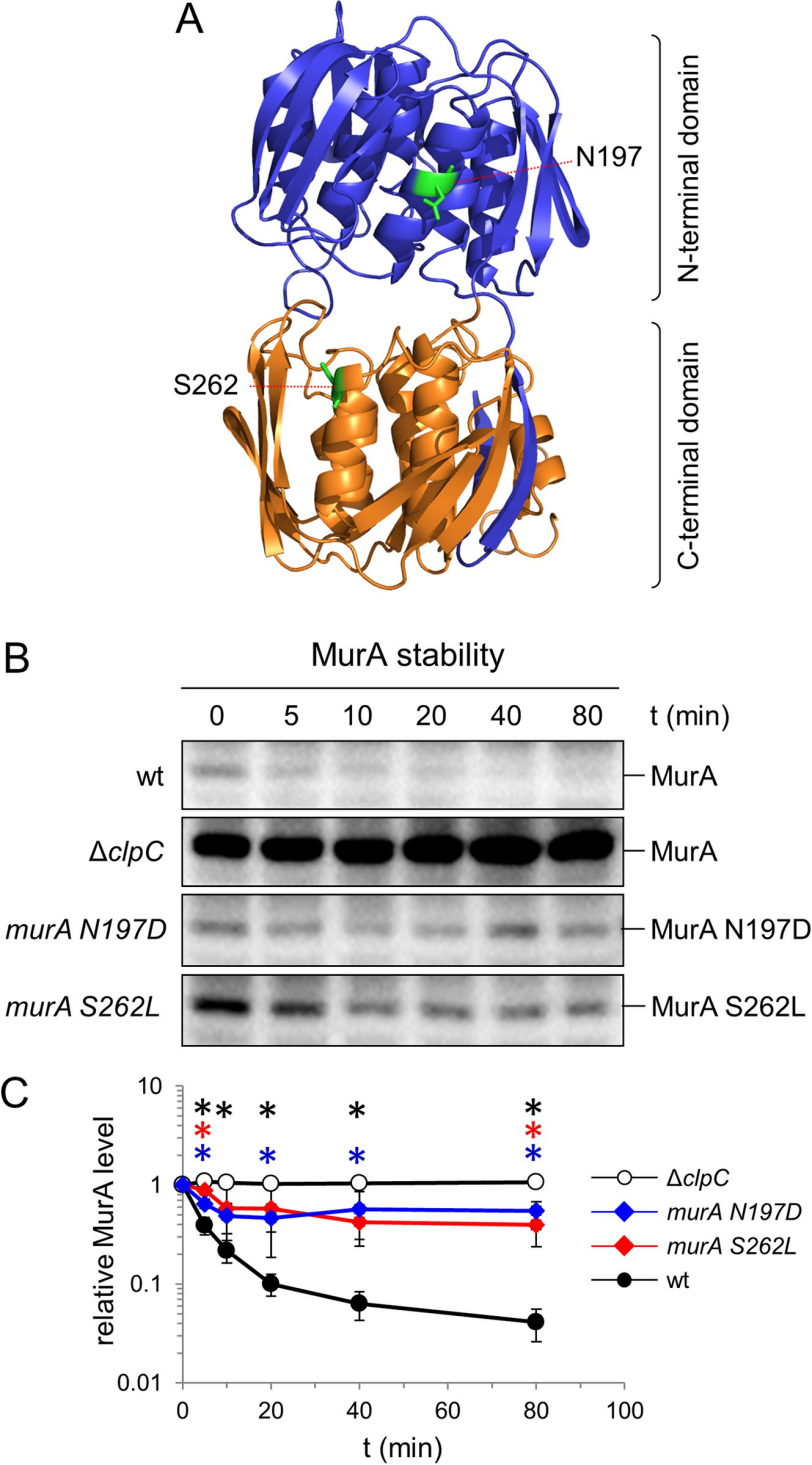
Effect of the N197D and S262L substitutions on MurA stability. (A) Structural model of the *L*. *monocytogenes* MurA gene (PDB code: 3R38) (70) with the N197 and S262 residues indicated (green coloring). (B) Western blots following MurA protein degradation *in vivo*. *L*. *monocytogenes* strains EGD-e (wt), LMJR138 (Δ*clpC*), LMSW155 (*murA S262L*) and LMSW156 (*murA N197D*) were cultivated to an OD_600_ of 1.0 and 100 μg/ml chloramphenicol was added to stop protein biosynthesis. Samples were taken before chloramphenicol addition and after different time points to analyze MurA levels. (C) MurA signals were quantified using ImageJ and average values and standard deviations are shown (n = 3). MurA levels of each strain before chloramphenicol addition (t = 0 min) were arbitrarily set to 1 and the remaining signal intensities at later time points are shown as relative values. Statistically significant differences (compared to wild type) are marked with asterisks (*P*<0.05, *t*-test with Bonferroni-Holm correction).

MurA proteins consist of two globular domains connected to each other. The UDP-Glc*N*Ac binding site and the catalytic center are located at the interface between these two domains [33]. The MurA residues replaced in the two *shg* suppressors are located outside the active site in helical regions exposed at the surface of the N-terminal (N197D) and the C-terminal (S262L) domain (Fig 2A). We assumed that these mutations would improve MurA activity to exert a similar suppressing effect on the Δ*gpsB* mutant as increased MurA levels. To test this, we generated strains that carry IPTG-inducible *murA* genes (wild type *murA* as well as S262L and N197D variants) at the *attB* plasmid integration site of their chromosomes but have their endogenous *murA* genes removed. Consistent with the essentiality of *murA* [27], all three strains required IPTG for growth in BHI broth (S2A Fig), when their pre-cultures were cultivated in the absence of IPTG. However, growth of strains LMSW140 (i*murA S262L*) and LMSW141 (i*murA N197D*) was similar to strain LMJR123 (i*murA*) in the presence of 1 mM IPTG (S2A Fig) and also at lower IPTG concentrations. Next, we measured the susceptibility of these strains to fosfomycin, a known inhibitor of MurA [34]. Fosfomycin sensitivity was measured in a disc diffusion assay on BHI agar plates, which allowed growth of the inducible *murA* strains even in the absence of IPTG due to background *murA* expression. Sensitivity of these strains towards fosfomycin was increased under these conditions compared to wild type due to MurA depletion, but effects of the N197D and S262L mutations did not become evident (S2B Fig).

To further test the possibility that the two *murA* mutations altered enzyme activity, wild type MurA, MurA N197D and MurA S262L were purified as Strep-tagged proteins to near homogeneity (S2C Fig) and their enzymatic activity was tested. This showed that the MurA N197D protein was as active as wild type MurA, whereas the activity of MurA S262L was reduced (S2D Fig). Taken together, these results rule out the possibility that increased MurA activities explain suppression of the Δ*gpsB* phenotype.

### The MurA N197D and S262L variants escape proteolytic degradation

Next, we wondered whether the two *murA* mutations affect proteolytic degradation of MurA. To begin with, this idea was tested in an experiment in which we titrated the expression level of *murA* and its N197D/S262L variants necessary for full suppression of the Δ*gpsB* growth defect. If any of these MurA variants is less prone to degradation and thus stabilized, then lower induction levels should be sufficient for suppression. In agreement with previous results [27], overexpression of wild type *murA* rescued growth of the Δ*gpsB* strain LMJR117 (S3A and S3B Fig), however, 0.5 mM was the minimal IPTG concentration leading to suppression. In contrast, ten-fold lower IPTG concentrations were sufficient for full suppression in the Δ*gpsB* strains LMS306 and LMS307 expressing IPTG-inducible *murA N197D* and *murA S262L* alleles, respectively, from their *attB* sites (S3C and S3D Fig). As this observation supports the idea of affected protein degradation, stability of MurA and its two variants was directly measured *in vivo*. To this end, the *gpsB* deletion in the two suppressor strains *shg19* (Δ*gpsB murA S262L*) and *shg21* (Δ*gpsB murA N197D*) was repaired by re-introduction of the native *gpsB* allele at the original locus to generate strains that carry the two *murA* mutations as the sole genetic changes. MurA degradation in the resulting strains LMSW155 (*murA S262L*) and LMSW156 (*murA N197D*) was then compared to wild type and the Δ*clpC* mutant. These strains were grown to an OD_600_ of 1.0 and 100 μg/ml chloramphenicol was added to block protein biosynthesis. MurA levels over time were then determined by Western blotting. As reported previously [24], MurA levels rapidly declined over time in wild type cells, but stayed stable in a Δ*clpC* mutant (Fig 2B and 2C), reflecting ClpCP-dependent proteolytic degradation of MurA. However, degradation of both MurA mutant proteins was significantly delayed (Fig 2B and 2C), which is in good agreement with their increased levels in the original *shg19* and *shg21* suppressors (Fig 1C). This shows that the MurA N197D and S262L variants escape proteolytic degradation by ClpCP.

### Effect of *murA* escape mutations on formation of MurA-ReoM complexes

**Fig 3:**
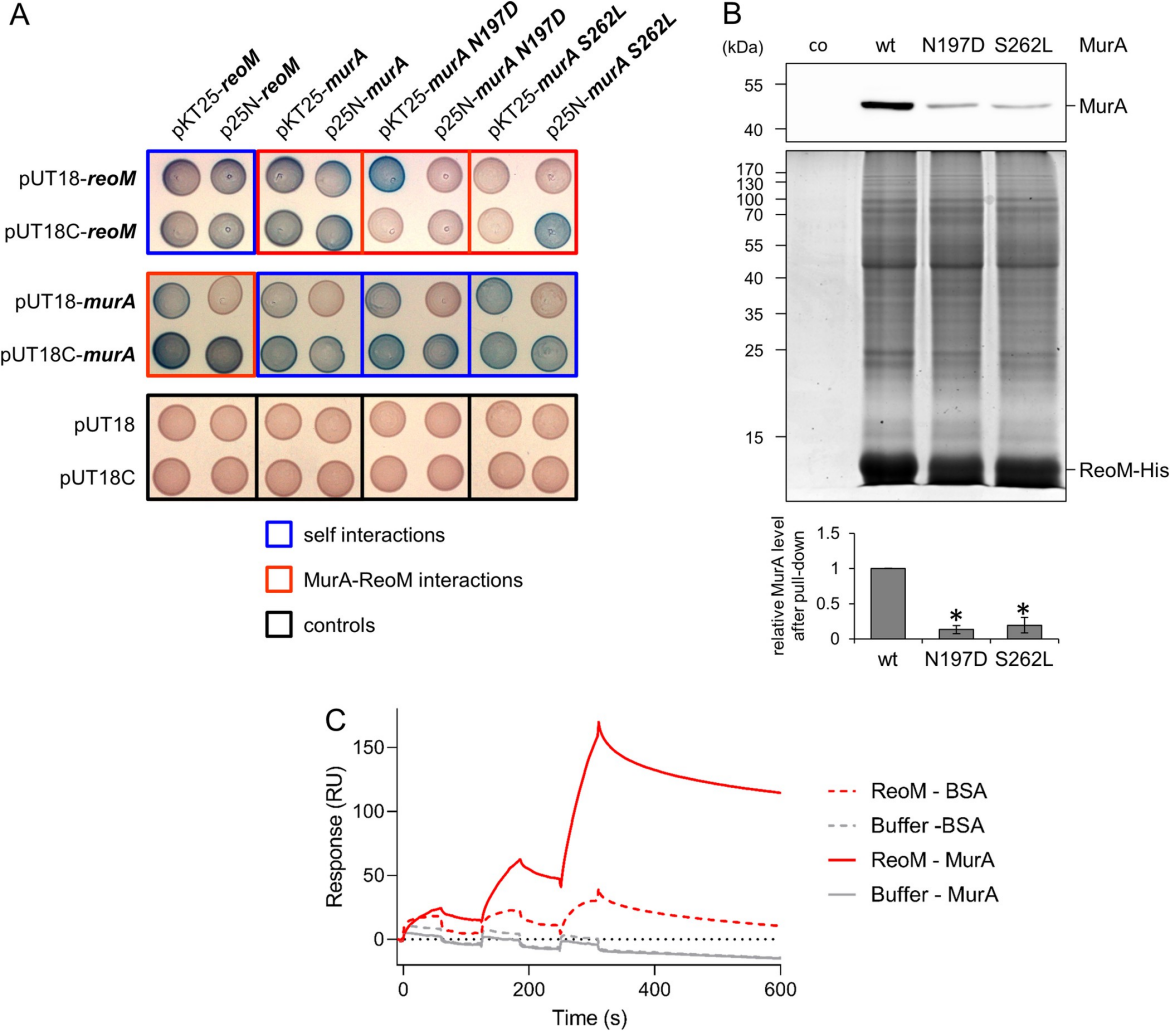
Effect of *murA* escape mutations on the interaction with ReoM. (A) Bacterial two hybrid assay to test the interaction of ReoM with MurA and its N197D and S262L variants. Variants of bacterial two hybrid vectors pUT18, pUT18C, pKT25 and p25N, which contain N- and C-terminal fusions of *reoM* and *murA* to the T18 and T25 fragments of the *B*. *pertussis* adenylate cyclase were co-transformed in *E*. *coli* BTH101 and selected on X-Gal containing agar (see Materials and Methods and Table 1 for more details). Blue coloring indicates an interaction between the tested proteins. Empty pUT18 and pUT18C vectors were included as negative controls. (B) Pull-down of MurA variants with ReoM-His as bait. *L*. *monocytogenes* strain EGD-e (co), and ReoM-His expressing strains LMPR1 (labelled “wt”), LMPR13 (“S262L”) and LMPR14 (“N197D”) were treated with formaldehyde prior to purification of ReoM-His. Purified eluates were analyzed for the presence of MurA by Western blotting (upper panel) or ReoM-His and copurifying proteins by SDS-PAGE (middle panel). MurA levels in the eluates were quantified by densitometry (lower panel). Average values and standard deviations from three independent experiments are shown. Asterisks mark statistically significant differences (*P*<0.01, *t*-test with Bonferroni-Holm correction). (C) Direct interaction of ReoM to immobilized MurA. Raw data sensorgrams for binding of ReoM or buffer only to BSA immobilized on reference flow cell 1 or to MurA immobilized on measurement flow cell 2. Shown are sensorgrams of 1:3 dilution series starting at 10 μM ReoM-dimer (MW 21 kDa).

Previously published bacterial two hybrid data showed that MurA interacts with ReoM, which–together with ReoY–could present MurA to ClpCP [24]. We wondered whether the MurA N197D and S262L mutants have lost the ability to form the complex with ReoM and tested this in the bacterial two hybrid system. As published before [24], B2H detects an interaction of MurA with ReoM (Fig 3A). However, the MurA N197D and S262L proteins have both lost their interaction with ReoM in several permutations, even though their interaction with wt MurA was unchanged (Fig 3A). To further validate this observation, we generated *murA* wt, N197D and S262L strains that produced his-tagged ReoM as a bait for pull-down analysis. We also deleted *clpC* in these strains to facilitate MurA detection after pull-down. The resulting strains were treated with formaldehyde to covalently crosslink interacting proteins prior to ReoM-His purification. Western blotting detected the presence of MurA in the eluates, indicating that MurA copurified with ReoM-His. Remarkably, MurA N197D (13±6% of wt MurA level) and S262L (19±11%) copurified to a lesser extent with ReoM-His (Fig 3B), even though their expression levels were similar to wt MurA in the Δ*clpC* background (S4 Fig). This confirms the idea that the two MurA variants could be stabilized because they do not longer bind to ReoM *in vivo*.

To study the interaction of ReoM with MurA even further, we switched to surface plasmon resonance. Binding was analysed in both orientations, *i*.*e*. with ReoM immobilized to the surface of the sensor chip and MurA as analyte in solution and *vice versa*. Specific binding was observed only when MurA was immobilized and ReoM was injected in solution (Fig 3C), but not in the opposite orientation. The interaction was characterized by a very slow association rate *k*_*a*_ of 5±1 × 10^2^ M^-1^s^-1^, but was relatively stable with a dissociation rate constant *k*_*d*_ of 4±2 × 10^−4^ s^-1^ leading to an overall affinity of 0.9±0.5 μM. Similar results were observed with MurA mutants N197D (K_D_ of 0.7±0.3 μM) and S262L (K_D_ of 0.8±0.4 μM, S5A, S5B, and S5C Fig). This shows that ReoM can bind MurA directly. However, this direct interaction is not sensitive to the two escape mutations. We assume that the interaction measured by SPR *in vitro* does not fully reflect the spectrum of interactions between ReoM with MurA that occurs *in vivo*.

### Escape mutations in *murA* affect peptidoglycan biosynthesis and growth

**Fig 4:**
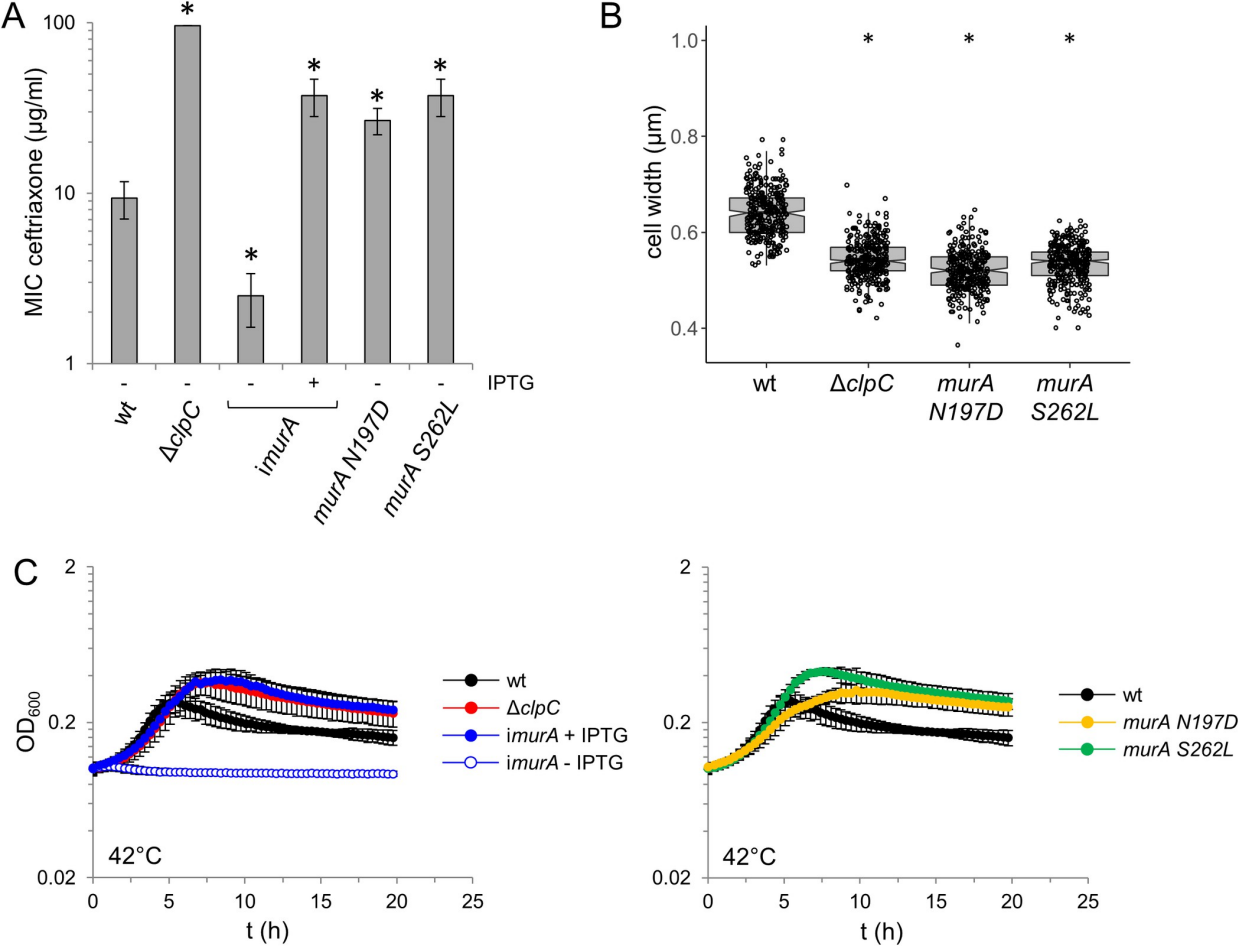
Effect of *murA* escape mutations on ceftriaxone resistance, morphology and growth at high temperature. (A) Minimal inhibitory concentrations (MIC) of ceftriaxone for *L*. *monocytogenes* strains EGD-e (wt), LMJR138 (Δ*clpC*), LMSW155 (*murA S262L*) and LMSW156 (*murA N197D*) as determined by E-tests. Strain LMJR123 (i*murA*) grown in the absence or presence of 1 mM IPTG was included for control. Average values and standard deviations are shown (n = 3). Asterisks mark statistically significant differences compared to wild type (*P*<0.05, *t*-test with Bonferroni-Holm correction). (B) Cell widths of *L*. *monocytogenes* strains EGD-e (wt), LMJR138 (Δ*clpC*), LMSW155 (*murA S262L*) and LMSW156 (*murA N197D*) during logarithmic growth in BHI broth at 37°C. Cells were stained with nile red and cell width of 300 cells per strain was measured. The experiment was repeated three times and one representative experiment is shown. Asterisks indicate significance levels compared to wild type (*—*P*<0.0001, *t*-test with Bonferroni-Holm correction). (C) Growth of the same set of strains as in panel A in BHI broth at 42°C. For clarity, growth curves were split into two diagrams. Average values and standard deviations from an experiment performed in triplicate are shown.

MurA was shown to be a key switch in regulation of PG biosynthesis and MurA levels strictly correlated with PG production and ceftriaxone resistance in *L*. *monocytogenes* [24,27]. Recent work in *B*. *subtilis* further confirmed this central role of MurA in PG biosynthesis regulation [35]. In order to test whether the two *murA* escape mutations influence PG production, their ceftriaxone resistance was tested and the Δ*clpC* and the i*murA* mutants were included as control. Ceftriaxone resistance was more than ten-fold higher in the Δ*clpC* mutant and inducer-dependent in the IPTG-controlled i*murA* strain as shown previously [24]. However, ceftriaxone resistance was also 3-4-fold higher in the two *murA* escape mutants, suggesting that stabilization of MurA in these two strains indeed caused stimulation of PG biosynthesis (Fig 4A). Fluorescence microscopy of nile red-stained cells also revealed changes in cellular morphology, as cells of the two *murA* escape and Δ*clpC* mutants were clearly thinner (Fig 4B), whereas cell length was not affected. Apparently, MurA stabilization also impairs maintenance of normal cell width.

Differences in growth of the *murA* escape mutants were not detected in BHI broth at 37°C, but a characteristic pattern that correlates well with the anticipated MurA expression levels became evident at 42°C. Here, the Δ*clpC* mutant grew to a higher optical density in stationary phase and a congruent growth curve was observed with the i*murA* strain in the presence of IPTG (Fig 4C). We have shown earlier that both strains highly overexpress MurA under these conditions [27]. Remarkably, both *murA* escape mutants also lead to higher stationary phase optical densities at 42°C (Fig 4C), suggesting that proteolytic degradation of MurA becomes important at higher temperature to limit bacterial growth. Analysis of the ultrastructure of the PG layer under these conditions revealed thicker PG layers in *murA N197D*, *murA S262L* and Δ*clpC* mutants, particularly at the poles (S6A, S6B, and S6C Fig). Moreover, polar PG thickness was found to be IPTG-dependent in a strain overexpressing *murA* (S6A and S6B Fig). This suggests that control of PG production through proteolytic stabilization of MurA controls listerial growth and influences PG ultrastructure, at least at higher temperature.

That the two *murA* escape mutations with their positive effects on growth and antibiotic resistance have not succeeded during evolution suggests that they are also associated with evolutionary disadvantages. Accordingly, the *murA N197D* and *S262L* mutants were found to grow slower in the presence of salt (S7A Fig) and were more sensitive against lysozyme (S7B Fig). Apparently, increased biomass yields that are caused by MurA overproduction comes at the price of increased sensitivity against different stressors.

### The *murA N197D* escape mutation uncouples viability and PG biosynthesis from PrkA signaling

**Fig 5:**
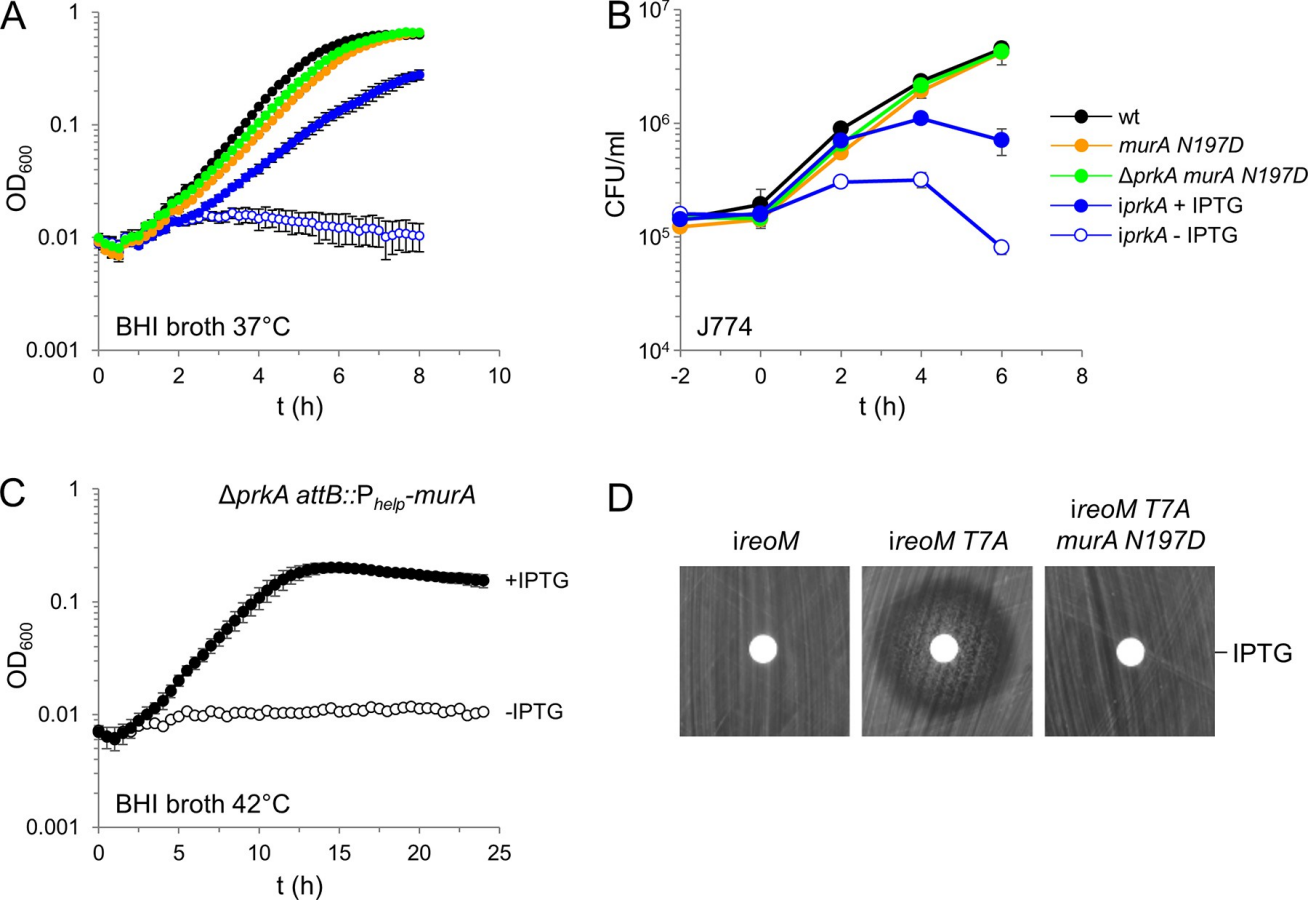
Suppression of lethal *prkA* and *reoM* phenotypes by *murA* mutations. (A) Growth of *L*. *monocytogenes* strains EGD-e (wt), LMSW84 (i*prkA*), LMSW156 (*murA N197D*) and LMS266 (Δ*prkA murA N197D*) in BHI broth at 37°C. Average values and standard deviations from technical replicates (n = 3) are shown. (B) Intracellular growth of strains EGD-e (wt), LMSW84 (i*prkA*), LMSW156 (*murA N197D*) and LMS266 (Δ*prkA murA N197D*) in J774 macrophages. The experiment was performed as triplicate and average values and standard deviations are shown. (C) MurA overexpression rescues the Δ*prkA* mutant. Growth of strain LMS272 (Δ*prkA attB*::P_*help*_*-murA*) in BHI broth ± 1 mM IPTG at 42°C. The experiment was carried out as triplicate and average values and standard deviations are shown. (D) The *murA N197D* mutation suppresses *reoM T7A* toxicity. Disc diffusion assay with filter discs loaded with 10 μl of a 1 M IPTG solution. Strains used were LMSW57 (i*reoM*), LMSW52 (i*reoM T7A*) and LMS273 (i*reoM T7A murA N197D*).

The *prkA* gene is essential in strain EGD-e because phosphorylated ReoM keeps ClpCP in control to prevent unregulated proteolysis of its essential substrate MurA [24]. Following with this model, MurA variants that escape proteolytic degradation through ClpCP should release the cell from PrkA dependency since ClpCP (even when overactive in the absence of PrkA and phosphorylated ReoM) does not longer accept such MurA variants as substrates. In order to test this hypothesis, we tried to delete *prkA* in *murA N197D* and *murA S262L* backgrounds, which never has been possible in the wild type background of *L*. *monocytogenes* EGD-e [24]. Similarly, the *prkA* gene could not be removed in the *murA S262L* background, presumably because of the reduced activity of MurA S262L. In contrast, *prkA* could be easily deleted in the *murA N197D* strain. The resulting Δ*prkA murA N197D* mutant was viable and grew like the wild type, whereas PrkA-depleted cells could not grow at all (Fig 5A). Moreover, the Δ*prkA murA N197D* mutant was viable inside macrophages and even grew intercellularly with a wild type-like growth rate. This was in complete contrast to PrkA-depleted cells (Fig 5B), suggesting that even the intracellular growth defect upon PrkA depletion may entirely be MurA-dependent.

We then tested whether overexpression of MurA would also rescue Δ*prkA* lethality. In fact, deletion of *prkA* turned out to be possible in strain LMJR116, in which a second copy of *murA* was expressed from an IPTG-dependent promoter. Growth of the resulting strain (LMS272) was not IPTG-dependent at 37°C, probably due to leakiness of the promoter controlling expression of the second *murA* copy. However, IPTG was required to support growth of this strain at 42°C (Fig 5C). The *clpC* gene is strongly induced at 42°C [36], leading to stimulated MurA degradation. This explains why IPTG-dependency of this strain was temperature dependent. Importantly, this experiment demonstrated that overexpression of MurA is sufficient to suppress the essentiality of *prkA*, which further supports the idea that control of MurA stability is the main purpose of PrkA signaling under the tested conditions.

If MurA is indeed the relevant subject of PrkA:ReoM signaling, then the *murA N197D* mutation should also overcome the toxicity of the phospho-ablative *reoM T7A* variant. We have shown in previous work that expression of *reoM T7A* is toxic to *L*. *monocytogenes* because MurA is rapidly degraded when ReoM cannot be phosphorylated [24]. To test this idea, a *murA N197D* strain was generated, in which expression of *reoM T7A* can be controlled by IPTG (strain LMS273) and susceptibility of this strain against IPTG was tested in a disc diffusion assay. While IPTG was clearly toxic for strain LMSW52 (i*reoM T7A*), strain LMS273 fully tolerated IPTG (Fig 5D). This further confirms that the main role of the PrkA:ReoM signaling axis is control of MurA stability, at least under the growth condition tested here.

**Fig 6:**
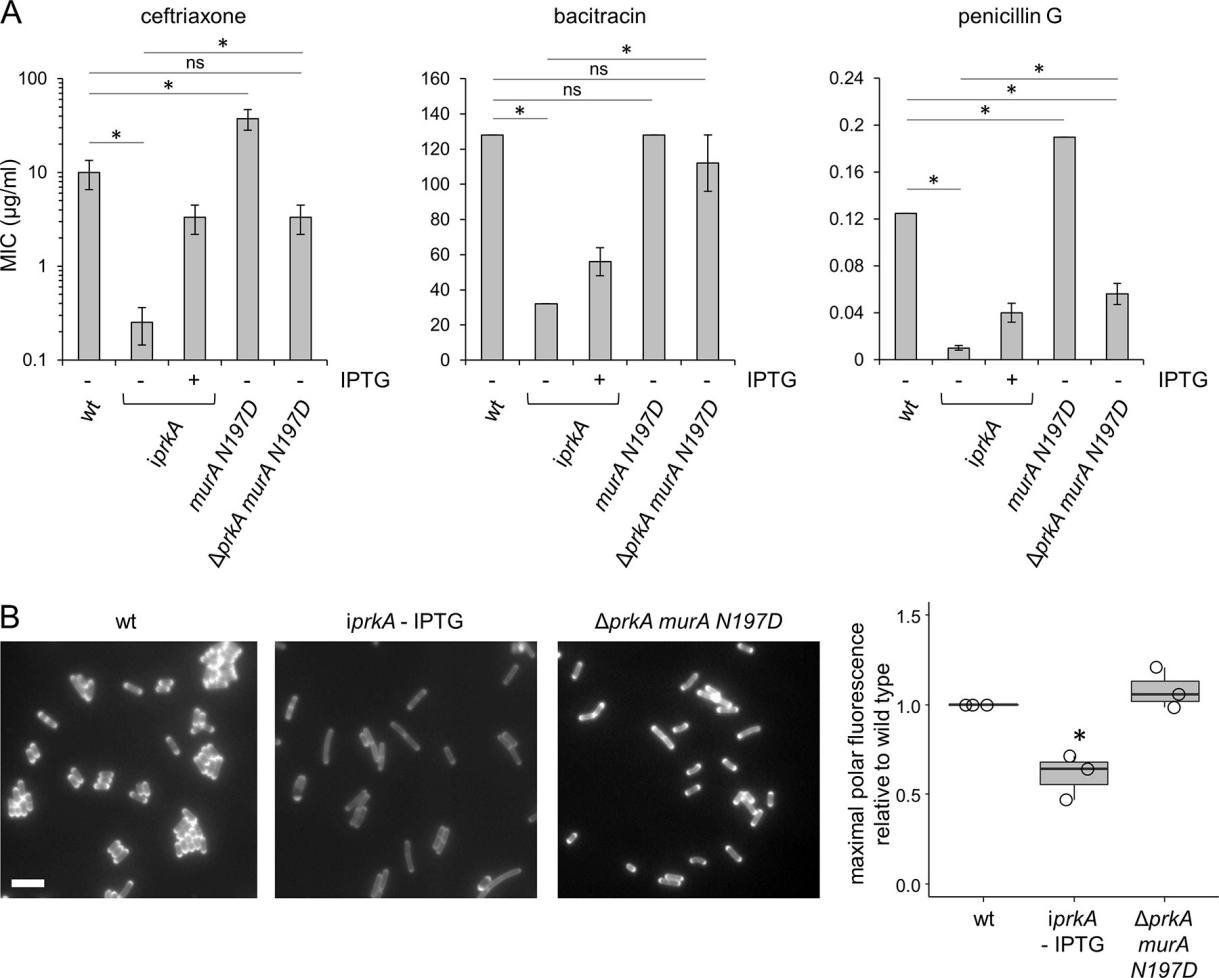
Suppression of *prkA* PG biosynthesis defects by *murA N197D*. (A) Minimal inhibitory concentrations of ceftriaxone, bacitracin and penicillin G for *L*. *monocytogenes* strains EGD-e (wt), LMSW84 (i*prkA*), LMSW156 (*murA N197D*) and LMS266 (Δ*prkA murA N197D*). Asterisks mark statistically significant differences compared to wild type or PrkA-depleted cells (n = 3, *P*<0.05, *t*-test with Bonferroni-Holm correction). (B) PG labelling in *prkA* and *murA* mutants. Micrographs showing HADA stained *L*. *monocytogenes* cells of strains EGD-e (wt), LMSW84 (i*prkA*) and LMS266 (Δ*prkA murA N197D*). Scale bar is 2 μm. Fluorescence intensity maxima at 20 cell poles per strain were quantified (rightmost panel). Average values from three experiments are shown. Asterisks indicate statistical significance (P<0.05, *t*-test with Bonferroni-Holm correction).

Interestingly, the extreme ceftriaxone sensitivity of PrkA-depleted cells did also not develop in the Δ*prkA murA N197D* strain (Fig 6A). Likewise, deletion of the *reoM*, *reoY* and *murZ* genes, which are all involved in control of MurA degradation, suppressed lethality and ceftriaxone susceptibility of the Δ*prkA* deletion–albeit to different degrees (S8 Fig). Moreover, enhanced bacitracin and penicillin G susceptibilities associated with PrkA depletion were also suppressed by the *murA N197D* mutation (Fig 6A). Since all these observations indicate restoration/activation of PG biosynthesis upon MurA stabilization, PG biosynthesis was analyzed directly by cell staining with the fluorescent D-alanine and PG precursor HADA [37]. As expected, PrkA-depleted cells showed reduced staining intensity compared to wildtype, particularly at the cell poles, and this effect was abolished in the Δ*prkA murAN197D* mutant (Fig 6B). Thus, PrkA not only becomes non-essential for viability and virulence, but also for PG biosynthesis in mutants, in which MurA escapes proteolytic degradation by ClpCP. Together with the results of Kelliher *et al*. [28], this suggests that PG biosynthesis is constitutively activated upon MurA stabilization.

To further verify our claims, we re-created the *murA N197D* mutation in the clean background of strain EGD-e. The resulting mutant (LMS308) showed the same ceftriaxone resistance as the *gpsB*-repaired *murA N197D* strain LMSW156 (S9A Fig). Furthermore, deletion of *prkA* in this background resulted in a viable strain (LMS309, S9B Fig), while deletion of *gpsB* yielded a strain (LMS310) that could grow at 42°C (S9C Fig). This proofs that we have not overlooked any other mutations in the genome sequence of the original *shg21* suppressor strain.

This extends previous conclusions on the relevance of ReoM for PrkA signaling [24,28] as it suggests that ReoM is specific for MurA and likely does not control the degradation of additional essential substrates in *L*. *monocytogenes* in a similar stringent manner.

### ClpC-dependent activation of PG biosynthesis requires PBP B3 and RodA3

Our data together with the data of Kelliher *et al*. [28] suggest that stabilization of MurA enhances PG formation. Moreover, they show that ceftriaxone resistance levels tightly correlate with the degree of MurA accumulation [24,27], suggesting that ceftriaxone resistance can be used as a proxy for PG biosynthesis. While most enzymes necessary for the cytoplasmic steps of PG biosynthesis are non-redundant, there is a high degree of redundancy in *L*. *monocytogenes* genes encoding PBPs and FtsW/RodA-like transglycosylases, required for the final steps outside the cell [38,39]. Inactivation of the class A PBPs had only mild effects on cephalosporin resistance in previous experiments [40], and were not further considered here. However, PBP B3, one out of three class B PBPs, substantially contributed to cephalosporin resistance [40,41]. Likewise, deletion of *rodA1* and *rodA2* only slightly affected cephalosporin resistance, whereas deletion of *rodA3* had a greater impact [39]. Thus, it seemed likely that PBP B3 and RodA3 cooperate to ensure activation of PG biosynthesis and ceftriaxone resistance when MurA accumulates.

**Fig 7:**
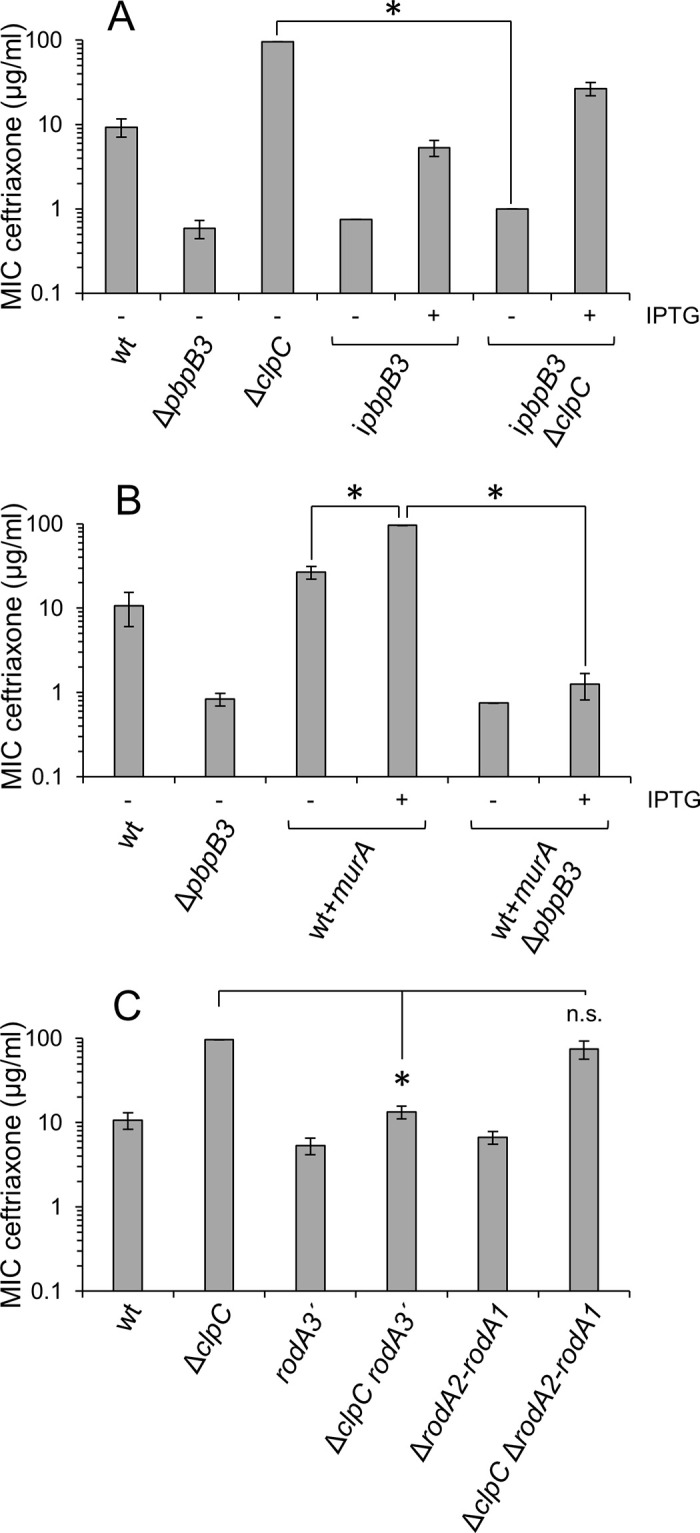
High ceftriaxone resistance upon MurA stabilization requires RodA3 and PBP B3. (A) Depletion of PBP B3 suppressed the increased ceftriaxone resistance of the Δ*clpC* mutant. Minimal inhibitory ceftriaxone concentrations of *L*. *monocytogenes* strains EGD-e (wt), LMJR41 (Δ*pbpB3*), LMJR138 (Δ*clpC*), LMMF1 (i*pbpB3*) and LMSW143 (i*pbpB3* Δ*clpC*) grown on agar plates ± 1 mM IPTG. (B) Deletion of *pbpB3* suppressed increased ceftriaxone resistance associated with MurA overexpression. Minimal inhibitory ceftriaxone concentrations for *L*. *monocytogenes* strains EGD-e (wt), LMJR41 (Δ*pbpB3*), LMJR116 (wt+*murA*) and LMSW168 (wt+*murA* Δ*pbpB3*) grown on agar plates ± 1 mM IPTG. (C) Inactivation of *rodA3* suppresses the increased ceftriaxone resistance of the Δ*clpC* mutant. Minimal inhibitory ceftriaxone concentrations of *L*. *monocytogenes* strains EGD-e (wt), LMJR138 (Δ*clpC*), LMS267 (*rodA3´*), LMS268 (Δ*clpC rodA3´*), LMSH67 (Δ*rodA2-rodA1*) and LMS270 (Δ*clpC* Δ*rodA2-rodA1*). MICs were determined using E-tests and average values and standard deviations calculated from three repetitions are shown. Asterisks indicate statistically significant differences compared to the Δ*clpC* mutant (*P*<0.01, *t*-test with Bonferroni-Holm correction).

In order to test this hypothesis, we determined the effect of *pbpB3* and *rodA3* mutations on the ceftriaxone resistance level of the Δ*clpC* mutant (MIC 96±0 μg/ml), which is strongly increased compared to wildtype (9.3±2.3 μg/ml, Fig 7A). As deletion of *pbpB3* was not possible in the Δ*clpC* background, *clpC* was deleted in an IPTG-inducible i*pbpB3* mutant. Depletion of PBP B3 in the i*pbpB3* mutant caused a similar reduction in ceftriaxone resistance in the absence of IPTG (MIC: 0.8±0 μg/ml) as observed in a Δ*pbpB3* strain (MIC: 0.6±0.1, Fig 7A). Remarkably, reduction of ceftriaxone resistance almost down to the level of a Δ*pbpB3* mutant was also observed in the i*pbpB3* Δ*clpC* strain, when grown in the absence of IPTG (1.0±0 μg/ml). MurA overexpression in the wild type background yielded the same ceftriaxone resistance level (MIC 96±0 μg/ml) as observed with the Δ*clpC* mutant and deletion of *pbpB3* also fully abrogated this effect (Fig 7B). This demonstrates that the increased ceftriaxone resistance levels of the Δ*clpC* mutant and after MurA overexpression are PBP B3-dependent.

Next, the *rodA2-rodA1* gene pair and the *rodA3* gene were inactivated in the Δ*clpC* mutant. While ceftriaxone resistance was only slightly affected by the Δ*rodA2-rodA1* deletion (MIC 6.7±1.2 μg/ml) compared to wildtype (10.7±2.4 μg/ml in this experiment), inactivation of *rodA3* had a somewhat stronger effect (5.3±1.2 μg/ml). More interestingly, the increased ceftriaxone resistance level of the Δ*clpC* mutant (96±0 μg/ml) was not affected by the Δ*rodA2-rodA1* deletion (74.7±18.5 μg/ml), but was reduced back almost to wildtype level when *rodA3* was inactivated (13.3±2.3 μg/ml, Fig 7C). All in all, this shows that PBP B3 and RodA3 collectively determine the increased ceftriaxone resistance of the Δ*clpC* mutant. This in turn suggests that PBP-FtsW and RodA could form a cognate pair and are of special importance for PG biosynthesis and ceftriaxone resistance, also under conditions leading to proteolytic stabilization of MurA.

## Discussion

Many substrates of PASTA-eSTKs have been identified in the different Gram-positive bacteria. Among these kinase substrates are proteins from various cellular pathways, but some functional classes are overrepresented: (i) Proteins acting in carbon and cell wall metabolism such as the HPr and YvcK proteins of *B*. *subtilis* and *L*. *monocytogenes* [28,42–44], (ii) regulatory proteins such as the WalR and GraR response regulators of *B*. *subtilis* and *Staphylococcus aureus*, respectively [45,46], and (iii) proteins acting in cell division, among which GpsB is one of the proteins consistently found as a PASTA-eSTK substrate in *B*. *subtilis*, *L*. *monocytogenes* and *Streptococcus pneumoniae* [28,47,48]. This diversity of substrates and their own-often pleiotropic-functions have impeded the identification of primary and the discrimination of less important kinase substrates. We previously have identified ReoM as a substrate of PrkA in *L*. *monocytogenes* EGD-e and could show that deletion of *reoM* suppresses loss of viability that results from PrkA depletion [24]. Moreover, expression of a phospho-ablative *reoM T7A* allele was toxic on its own and thus phenocopied the absence of PrkA [24]. These tight geno- and phenotypic relations between the PrkA kinase and ReoM, which is only one out of 23 known PrkA substrates in *L*. *monocytogenes* [28], suggested that ReoM must be a relevant kinase target. We here further support this conclusion by our observation that *prkA* even can be deleted in a Δ*reoM* background, again indicating that the phosphorylation of ReoM is the particular PrkA-dependent phosphorylation event that is crucial for viability. Remarkably, Kelliher *et al*. reported that *prkA* is non-essential in *L*. *monocytogenes* strain 10403S [28] and discuss strain-specific differences as an explanation. In fact, *L*. *monocytogenes* strains EGD-e and 10403S are relatively distinct with respect to the population structure of the species *L*. *monocytogenes* and even belong to different molecular serogroups (EGD-e: IIc, 10403S: IIa) [49]. Thus, differences in the stringency of MurA degradation by ClpCP, in expression of the genes acting in the ReoM/ClpCP/MurA axis or even in the relative expression of *murA* and its paralogue *murZ* may account for this difference. Despite this discrepancy and consistent with our findings, *reoM* deletion suppressed all *prkA* associated phenotypes also in strain 10403S [28].

How ReoM stimulates MurA degradation is presently not known, but it may act as a ClpCP activator or as an adaptor, which presents substrates to the protease complex. ReoM could either be specific for MurA or could also control the stability of other proteins. Our observation that MurA variants exist that escape proteolytic degradation, further supports the idea that MurA is a protease substrate even though this has never been confirmed *in vitro* [24,27,30]. Moreover, the observations that their amino acid substitutions are located on the MurA surface and impair the interaction with ReoM would be consistent with the adaptor hypothesis. An interesting aspect is the observation that the escape mutations disturb formation of ReoM:MurA complexes *in vivo* (even in a heterologous system) but not *in vitro*. We cannot explain this at the moment, but speculate that effects that occur during translation of MurA, for which SPR is insensitive, could be a possible reason for this discrepancy.

**Fig 8:**
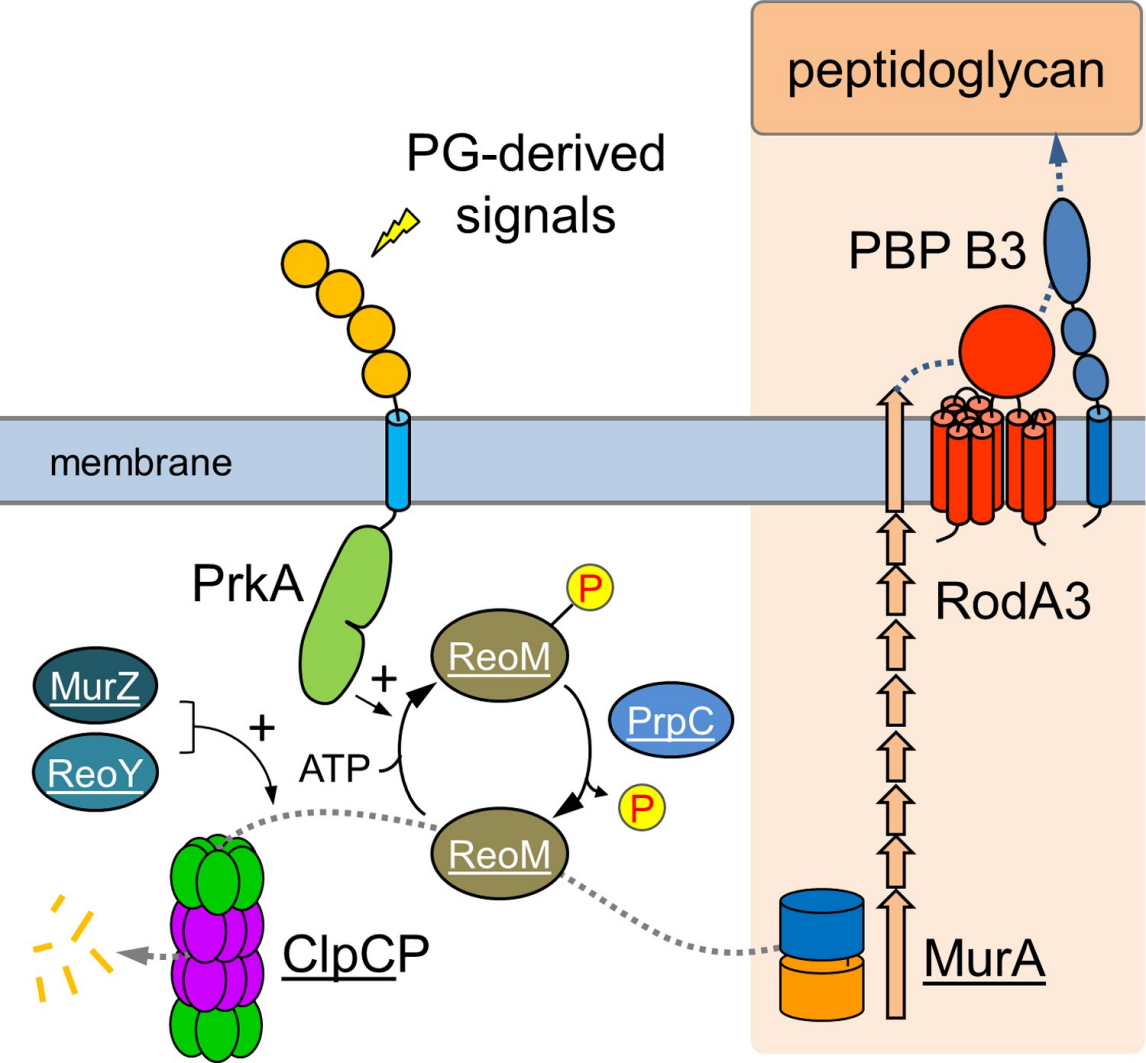
Control of *L*. *monocytogenes* PG biosynthesis through PrkA signaling. PrkA phosphorylates ReoM upon activation by PG-derived signals such as lipid II. P-ReoM no longer supports/activates ClpCP-dependent MurA degradation so that MurA can accumulate and PG biosynthesis can take place. Activation of PG biosynthesis under conditions leading to PrkA activation and MurA accumulation requires RodA3 and PBP B3. Underlined proteins have been identified as suppressors of the Δ*gpsB* mutant, which is affected in PG biosynthesis, in this and previous work [24,27]. Image with modifications from [74].

A central result of our study is the finding that one of the *murA* escape mutations (N197D) suppressed *prkA* essentiality under standard growth conditions and during intracellular growth in macrophages, to which *prkA* mutants are specifically sensitive [44]. This particular *murA* mutation does not alter enzymatic activity and only rescues MurA from proteolytic degradation. It also overcomes the lethality of the *reoM T7A* allele, the expression of which normally would lead to rapid MurA degradation. These findings provide a genetic answer to the question whether other *L*. *monocytogenes* proteins exist, the stability of which depends on ReoM phosphorylation: As uncoupling of MurA from PrkA-dependent control of ReoM-mediated ClpCP activation restores otherwise lethal *prkA* and *reoM* phenotypes, we have to conclude that MurA is not only the main substrate or target of ReoM, but also that control of MurA stability is the main purpose of PrkA-mediated signaling in *L*. *monocytogenes* during normal laboratory growth and inside macrophages. This conclusion is further substantiated by the observation that artificial MurA overexpression also rescues the lethal Δ*prkA* phenotype. According to our present model, PrkA gets activated during cell wall stress and phosphorylation of ReoM limits MurA degradation (Fig 8). Our data suggest that PG biosynthesis is activated when MurA accumulates. How PG biosynthesis activation under these conditions alters the chemical structure of the sacculus is not known, but a thicker cell wall is produced, especially at the poles, and this occurs concomitantly with an increase of ceftriaxone resistance. *L*. *monocytogenes* PrkA presumably localizes to the cell division septum as reported for the PASTA kinases of *B*. *subtilis*, *S*. *pneumoniae* and *S*. *aureus* [17,47,50]. Such a possible septal localization of PrkA could be related to the polar PG thickening in *murA* (this work), *reoM* and *reoY* mutants [24]. In all these mutants, MurA escapes degradation and if this occurs primarily at those subcellular sites, at which PrkA is enriched (*i*. *e*. the septum), then thicker PG cross walls and later thicker polar PG layers might be a possible consequence. That the *murA* escape mutants are more susceptible to lysozyme despite their thicker PG might be explained by imbalances between increased PG biosynthesis and unaltered PG modification. PG modifying reactions such as N-deacetylation and O-acetylation are the main determinants of lysozyme resistance in *L*. *monocytogenes* [51,52] and increased lysozyme resistance could occur when PG modification does not keep pace with PG biosynthesis.

As a second important result of our study we could show that the high level of ceftriaxone resistance upon MurA stabilization depends on RodA3 and PBP B3, which may constitute a cognate glycosyltransferase/transpeptidase pair (Fig 8). In our current model, the PrkA→ReoM/ReoY→MurA/ClpCP→RodA3/PBP B3 cascade would represent a closed homeostatic circuit that senses lipid II (by PrkA) to control its production (through control of MurA stability) and consumption (by RodA3/PBP B3) (Fig 8). The genes in this pathway collectively determine the intrinsic resistance of *L*. *monocytogenes* to cephalosporins [24,39–41,53] and are conserved in several other Gram-positive bacteria (S10 Fig). In *Enterococcus faecalis*, most of the corresponding homologues were shown to maintain the high intrinsic cephalosporin resistance level of this organism [29,54–57], and deletion of *pbpC*, which encodes the PBP B3 homologue of *B*. *subtilis* (S10 Fig), was also necessary for cephalosporin resistance [58]. Moreover, inactivation of Stk1, the PASTA-eSTK of *S*. *aureus*, sensitizes methicillin resistant *S*. *aureus* (MRSA) against β-lactams including oxacillin and this is overcome by *reoM* and *reoY* deletions [28,59]. Interestingly, MecA, which represents the major methicillin resistance determinant of MRSA [60], is the equivalent homologue of *L*. *monocytogenes* PBP B3 (S10 Fig) [8], suggesting that methicillin resistance in MRSA is regulated in an analogous way. Lastly, the PrkA signaling cascade lacks ReoY and a homologue of PBP B3 in *S*. *pneumoniae*, which is typically susceptible to penicillins. This raises the interesting possibility that this cascade generally supports a specific type of PG biosynthesis or repair, which confers a higher resistance against cephalosporins and other β-lactams. In which way PG biosynthesis is altered under such conditions is not known, but PG formation at specific subcellular sites or generation of PG with particular crosslinking rates or with a certain tertiary structure would be conceivable options. Further studies, also including other bacterial species, would have to be performed to test such hypotheses.

## Materials and methods

### Bacterial strains and growth conditions

Table 1 lists all strains used in this study. *L*. *monocytogenes* strains were routinely cultivated in brain heart infusion (BHI) broth or on BHI agar plates. Growth curves were recorded in 96 well microplate readers. Antibiotics and supplements were added when required at the following concentrations: erythromycin (5 μg/ml), kanamycin (50 μg/ml), X-Gal (100 μg/ml) and IPTG (as indicated). *Escherichia coli* TOP10 was used as standard host for all cloning procedures [61].

**Table 1:** Plasmids and strains used in this study.

name	relevant characteristics	source*/ reference	
**Plasmids**			
pET11a	*bla* P_*T7*_ *lacI*	Novagen	
pIMK2	P_*help*_ *neo*	[62]	
pMAD	*bla erm bgaB*	[32]	
pUT18	*bla* P_*lac*_*-cya(T18)*	[63]	
pUT18C	*bla* P_*lac*_*-cya(T18)*	[63]	
pJR24	*bla erm bgaB* Δ*pbpB3 (lmo0441)*	[38]	
pJR67	*bla erm bgaB* Δ*murA (lmo2526)*	[27]	
pJR82	P_*help*_*-lacO-murA lacI neo*	[27]	
pJR101	*kan* P_*lac*_*-cya(T25)-reoM*	[24]	
pJR102	*kan* P_*lac*_*-reoM-cya(T25)*	[24]	
pJR103	*bla* P_*lac*_*-reoM-cya(T18)*	[24]	
pJR104	*bla* P_*lac*_*-cya(T18)-reoM*	[24]	
pJR116	*kan* P_*lac*_*-cya(T25)-murA*	[24]	
pJR117	*kan* P_*lac*_*-murA-cya(T25)*	[24]	
pJR118	*bla* P_*lac*_*-murA-cya(T18)*	[24]
pJR119	*bla* P_*lac*_*-cya(T18)-murA*	[24]
pJR126	*bla erm bgaB* Δ*reoM (lmo1503)*	[24]	
pJR127	*bla erm bgaB* Δ*clpC (lmo0232)*	[27]	
pSAH62	*bla erm bgaB* Δ*rodA2-rodA1* (*lmo2428-2427*)	[64]	
pSAH67	*bla erm bgaB ´rodA3´* (*lmo2687*)	[64]	
pSH242	*bla erm bgaB gpsB* (*lmo1888*) region	[26]	
pSH246	*bla erm bgaB* Δ*gpsB*	[26]	
pSW29	P_*help*_*-lacO-reoM T7A lacI neo*	[24]	
pSW36	*bla erm bgaB* Δ*prkA (lmo1820)*	[24]	
pSW37	*bla erm bgaB* Δ*prpC (lmo1821)*	[24]	
pJD16	P_*help*_*-reoM-his neo*	this work	
pSH571	*bla erm bgaB murA N197D*	this work	
pSW52	*bla P*_*T7*_ *-murA-strep lacI*	this work	
pSW61	*bla P*_*T7*_ *-murA S262L-strep lacI*	this work	
pSW62	*bla P*_*T7*_ *-murA N197D-strep lacI*	this work	
pSW63	P_*help*_*-lacO-murA S262L lacI neo*	this work	
pSW64	P_*help*_*-lacO-murA N197D lacI neo*	this work	
pSW66	*kan* P_*lac*_*-cya(T25)-murA S262L*	this work	
pSW67	*kan* P_*lac*_*-murA S262L-cya(T25)*	this work	
pSW68	*kan* P_*lac*_*-cya(T25)-murA N197D*	this work	
pSW69	*kan* P_*lac*_*-murA N197D-cya(T25)*	this work	
***L*. *monocytogenes* strains**			
EGD-e	wild-type, serovar 1/2a strain	[65]	
LMJR19	Δ*gpsB*	[26]	
LMJR41	Δ*pbpB3*	[38]	
LMJR104	Δ*murZ*	[27]	
LMJR116	*attB*::P_*help*_*-lacO-murA lacI neo*	[27]	
LMJR117	Δ*gpsB attB*::P_*help*_*-lacO-murA lacI neo*	[27]	
LMJR123	Δ*murA attB*::P_*help*_*-lacO-murA lacI neo*	[27]	
LMJR138	Δ*clpC*	[27]	
LMJR139	Δ*gpsB* Δ*clpC*	[27]	
LMMF1	Δ*pbpB3 attB*::P_*help*_*-lacO-pbpB3 lacI neo*	[41]	
LMSH67	Δ*rodA2-rodA1*	[64]	
LMSW30	Δ*reoM*	[24]	
LMSW32	Δ*reoY*	[24]	
LMSW52	Δ*reoM attB*::P_*help*_*-lacO-reoM T7A lacI neo*	[24]	
LMSW57	Δ*reoM attB*::P_*help*_*-lacO-reoM lacI neo*	[24]	
LMSW84	Δ*prkA attB*::P_*help*_*-lacO-prkA lacI neo*	[24]	
*shg19*	Δ*gpsB murA S262L*	this work	
*shg21*	Δ*gpsB murA N197D*	this work	
*shg24*	Δ*gpsB prpC N83S L125F*	this work	
*shg32*	Δ*gpsB prpC*^*1-109*^ ^*#*^	this work	
*shg42*	Δ*gpsB prpC*^*1-73*^ ^*#*^	this work	
*shg47*	Δ*gpsB prpC G39S*	this work	
*shg55*	Δ*gpsB prpC*^*1-44*^ ^*#*^	this work	
LMPR1	Δ*clpC attB*::P_*help*_*-reoM-his neo*	pJD16 → LMJR138	
LMPR8	*murA S262L attB*::P_*help*_*-reoM-his neo*	pJD16 → LMSW155	
LMPR9	*murA N197D attB*::P_*help*_*-reoM-his neo*	pJD16 → LMSW156	
LMPR13	Δ*clpC murA S262L attB*::P_*help*_*-reoM-his neo*	pJR127 ↔ LMPR8	
LMPR14	Δ*clpC murA N197D attB*::P_*help*_*-reoM-his neo*	pJR127 ↔ LMPR9	
LMS266	Δ*prkA murA N197D*	pSW36 ↔ LMSW156	
LMS267	*rodA3´*	pSAH67 → EGD-e	
LMS268	Δ*clpC rodA3´*	pSAH67 → LMJR138	
LMS270	Δ*clpC* Δ*rodA2-rodA1*	pSAH62 ↔ LMJR138	
LMS271	Δ*reoM murA N197D*	pJR126 ↔ LMSW156	
LMS272	Δ*prkA attB*::P_*help*_*-lacO-murA lacI neo*	pSW36 ↔ LMJR116	
LMS273	Δ*reoM attB*::P_*help*_*-lacO-reoM T7A lacI neo murA N197D*	pSW29 → LMS271	
LMS306	Δ*gpsB attB*::P_*help*_*-lacO-murA S262L lacI neo*	pSW63 → LMJR19	
LMS307	Δ*gpsB attB*::P_*help*_*-lacO-murA N197D lacI neo*	pSW64 → LMJR19	
LMS308	*murA N197D*	pSH57 ↔ EGD-e	
LMS309	Δ*prkA murA N197D*	pSW36 ↔ LMS308	
LMS310	Δ*gpsB murA N197D*	pSH246 ↔ LMS308	
LMSW135	Δ*gpsB* Δ*prpC*	pSW37 ↔ LMJR19	
LMSW136	*attB*::P_*help*_*-lacO-murA S262L lacI neo*	pSW63 → EGD-e	
LMSW137	*attB*::P_*help*_*-lacO-murA N197D lacI neo*	pSW64 → EGD-e	
LMSW140	Δ*murA attB*::P_*help*_*-lacO-murA S262L lacI neo*	pJR67 ↔ LMSW136	
LMSW141	Δ*murA attB*::P_*help*_*-lacO-murA N197D lacI neo*	pJR67 ↔ LMSW137	
LMSW143	Δ*pbpB3 attB*::P_*help*_*-lacO-pbpB3 lacI neo* Δ*clpC*	pJR127 ↔ LMMF1	
LMSW144	Δ*prkA* Δ*reoY*	pSW36 ↔ LMSW32	
LMSW145	Δ*prkA* Δ*murZ*	pSW36 ↔ LMJR104	
LMSW146	Δ*prkA* Δ*reoM*	pSW36 ↔ LMSW30	
LMSW155	*murA S262L*	pSH242 ↔ *shg19*	
LMSW156	*murA N197D*	pSH242 ↔ *shg21*	
LMSW168	Δ*pbpB3 attB*::P_*help*_*-lacO-murA lacI neo*	pJR24 ↔ LMJR116	

* The arrow (→) stands for a transformation event and the double arrow (↔) indicates gene deletions obtained by chromosomal insertion and subsequent excision of pMAD plasmid derivatives (see Materials and methods for details).

^#^ Numbers indicate amino acid positions.

### General methods, manipulation of DNA and oligonucleotide primers

Standard methods were used for transformation of *E*. *coli*, isolation of plasmid DNA and chromosomal DNA isolation [61]. Transformation of *L*. *monocytogenes* was carried out by electroporation as described by others [62]. The manufacturer´s instructions were followed for restriction and ligation of DNA. All primer sequences are listed in Table 2.

**Table 2:** Oligonucleotides used in this study.

name	sequence (5´→3´)
JD13	CGCGCGCTGCAGGAAAGATCAAACAATGTTTTACAACT
JD14	CGCGCGGTCGACTCAATGGTGATGGTGGTGATGGTGATGGTGATGTTTCTCACCAATTTCGTTATTTTTC
JD25	AACCCATGGATTCAAAAGATCAAACAATGTTTTACAACTTCG
JD26	TTTGAATCCATGGGTTTCACTCTCCTTCTAC
SHW948	CTAGACAGATCTATCGATGCATGCCATGGTTGATATTTTAGGGTGATAAGTGG
SHW949	GCCTCGCGTCGGGCGATATCGGATCCGTACCTAAATCGATACCAACATC
SHW950	ACTTGGCAGATTTCCTTAACCAAATGGGTGCTAG
SHW951	TTAAGGAAATCTGCCAAGTCAACAATTTCAGGTTC
SW130	GCGCGCACTAGTTTGGAAAAAATTATTGTACGCGGTGG
SW131	CGCGCGCTCGAGTTATTTTTCGAACTGCGGGTGGCTCCAGAATAAAGACGCTAAGTTTGTTACATCG
SW150	GCAATTAACAGACTAATATGTTCAGGAACTGCATCTTC
SW151	CATATTAGTCTGTTAATTGCTAAACTTGAAGAAATGGGC
SW152	GTTAAGGAAGTCTGCCAAGTCAACAATTTCAGGTTC
SW153	GACTTGGCAGACTTCCTTAACCAAATGGGTGCTAG

### Construction of plasmids and strains

Plasmid pSW52 was constructed for overexpression of Strep-tagged MurA in *E*. *coli*. To this end, the *murA* open reading frame was amplified with primers SW130/SW131 and cloned into pET11a using SpeI/XhoI. The S262L and N197D mutations were then introduced into pSW52 by quikchange mutagenesis using the primer pairs SW150/SW151 and SW152/SW153, respectively. The same primers were used to introduce both *murA* mutations into plasmid pJR82 by quikchange mutagenesis, yielding plasmids pSW63 and pSW64 for inducible expression of *murA S262L* and *murA N197D*, respectively, in *L*. *monocytogenes*. Likewise, these primers were used to introduce both mutations into the *murA* gene of the bacterial two hybrid vectors pJR116 and pJR117.

Plasmid pSH571 was constructed for introduction of the *murA N197D* mutation into the genome. For this purpose, *murA* fragments up- and downstream of N197 were amplified with primers SHW948/SHW951 and SHW950/SHW949, respectively, and joined by splicing by overlapping extension PCR (SHW950 and SHW951 introduced the N197D mutation). The resulting fragment was inserted into pMAD by restriction free cloning.

Plasmid pJD16 was generated for *reoM-his* expression in *L*. *monocytogenes*. To this end, *reoM* was amplified using JD13/JD14 as the primers (the reverse primer introduced a His-10 tag) and cloned into pIMK2 using PstI/SalI. An unwanted sequence that arose during cloning was then removed from the *reoM* N-terminus by restriction free cloning using the oligonucleotides JD25/JD26.

Derivatives of pIMK2 and pIMK3 were introduced into *L*. *monocytogenes* strains by electroporation and clones were selected on BHI agar plates containing kanamycin. Plasmid insertion at the *attB* site of the tRNA^Arg^ locus was verified by PCR. Plasmid derivatives of pMAD were transformed into the respective *L*. *monocytogenes* recipient strains and genes were deleted as described elsewhere [32]. All gene deletions and insertions were confirmed by PCR. Plasmid pSAH67, designed for insertional disruption of *rodA3*, was transformed into *L*. *monocytogenes* and transformants were selected on BHI agar plates containing erythromycin at 30°C. Plasmid insertion into the chromosomal *rodA3* gene was enforced by streaking the transformants to single colonies on BHI agar plates containing erythromycin at 40°C. Disruption of *rodA3* was confirmed by PCR.

### Antibiotic susceptibility testing

Minimal inhibitory concentrations were determined as described previously using E-test strips with a ceftriaxone or bacitracin concentration range of 0.016–256 μg/ml or a penicillin G concentration range of 0.002–32 μg/ml (all from Bestbion^dx^, Germany) [38]. Susceptibility against fosfomycin was tested in a disc diffusion assay using filter discs loaded with 8 μl of a 20 mg/ml fosfomycin solution. For this purpose, *L*. *monocytogenes* colonies, grown on BHI agar plates, were resuspended in BHI broth and used to swab-inoculate BHI agar plates. Filter discs were spotted on BHI agar plates and incubated over night at 37°C to measure the inhibition zone diameter the next day.

### Bacterial two hybrid experiments

Plasmids carrying genes fused to T18- or the T25-fragments of the *Bordetella pertussis* adenylate cyclase (Table 1) were co-transformed into *E*. *coli* BTH101 [63] and transformants were selected on LB agar plates containing ampicillin (100 μg ml^-1^), kanamycin (25 μg ml^-1^), X-Gal (0.004%) and IPTG (0.1 mM). Agar plates were photographed after 48 h of incubation at 30°C.

### Genome sequencing

Chromosomal DNA for genome sequencing was isolated using the GenElute Bacterial Genomic DNA Kit (Sigma-Aldrich). Libraries were generated from 1 ng genomic DNA by using the Nextera XT DNA Library Prep Kit (Illumina). Sequencing was performed using a MiSeq Reagent Kit v3 cartridge (600-cycle kit) on a MiSeq benchtop sequencer in paired-end mode (2 x 300 bp). Reads were mapped to the *L*. *monocytogenes* EGD-e reference genome (NC_003210.1) [65] in Geneious (Biomatters Ltd.) to identify single nucleotide polymorphisms using the Geneious SNP finder tool. Genome sequences of *gpsB* suppressor strains were deposited at ENA under project accession number PRJEB47255.

### Isolation of cellular proteins and Western blotting

For protein isolation, cells from a 20 ml culture volume were harvested by centrifugation, washed with ZAP buffer (10 mM Tris/HCl pH7.5, 200 mM NaCl), resuspended in 1 ml ZAP buffer also containing 1 mM PMSF and disrupted by sonication. Cellular debris was removed by centrifugation and the supernatant was considered as total cellular protein extract. Aliquots of these protein samples were separated by SDS polyacrylamide gel electrophoresis (PAGE) and transferred onto positively charged polyvinylidene fluoride membranes employing a semi-dry transfer unit. DivIVA and MurA were detected by polyclonal rabbit antisera recognizing *B*. *subtilis* MurAA [66] and DivIVA [67] as the primary antibodies, respectively. An anti-rabbit immunoglobulin G conjugated to horseradish peroxidase was used as the secondary antibody and detected using the ECL chemiluminescence detection system (Thermo Scientific) in a chemiluminescence imager (Vilber Lourmat).

### *In vivo* formaldehyde crosslinking and pull-down

*In vivo* crosslinking of protein complexes using formaldehyde, purification of His-tagged bait proteins using MagneHis magnetic beads and protein decrosslinking by heat treatment were performed according to a previously published protocol [68]. Decrosslinked samples were separated by SDS-PAGE and either stained using a colloidal Coomassie stain or analyzed by Western blotting.

### Protein purification

Proteins were overproduced in *E*. *coli* BL21. Overexpression strains were cultivated in 1 l LB broth (containing 100 μg/ml ampicillin) at 37°C and 250 rpm. Protein expression was induced by addition of 1 mM IPTG at OD_600_ = 0.5. Cultivation was continued over night at 16°C and 200 rpm. Cells were harvested by centrifugation (11.325 x g, 10 min, 4°C) and the cell pellet was washed once in 20 ml ZAP buffer. Afterwards, the cell pellet was resuspended in 20 ml ZAP buffer containing 1 mM PMSF. Cells were disrupted using an EmulsiFlex C3 homogenizer (Avestin Europe GmbH), cell debris was removed by centrifugation (4.581 x g, 10 min, 4°C) and the lysate was cleared in an additional passage through a filter (0.45 μm pore size). Strep-tagged proteins were purified using affinity chromatography and Strep-Tactin Sepharose (IBA Lifesciences, Germany) according to the manufacturer’s instructions. Fractions containing purified proteins were pooled, aliquoted and stored at -20°C.

### MurA activity assay

An assay monitoring phosphate release from phosphoenol-pyruvate (PEP) was used for determination of MurA activity [69]. For this purpose, 5 μg of purified MurA protein were mixed with 10 mM uridine 5′-diphospho-*N*-acetylglucosamine (UDP-Glc*N*Ac, Sigma-Aldrich) in a reaction buffer containing 100 mM Tris/HCl pH8.0 and 150 mM NaCl (final volume: 50 μl) and preincubated at 37°C for 15 min. The reaction was started by addition of 5 μl 10 mM PEP (Sigma-Aldrich) and stopped by addition of 800 μl staining solution after different time intervals. The staining solution was freshly prepared from 10 ml ammonium molybdate solution (4.2 g in 100 ml 4 M HCl), 30 ml malachite green solution (225 mg malachite green in 500 ml H_2_O) and 10 μl Triton X-100. Absorption was measured at λ = 660 nm, corrected for background in the absence of UDP-Glc*N*Ac and used to calculate the amount of released phosphate using a standard curve generated with solutions with different phosphate concentrations.

### Surface plasmon resonance

Binding kinetics between ReoM and MurA were analysed by surface plasmon resonance (SPR) measurements at 25°C using HBS-EP+ as running buffer (10 mM HEPES, 150 mM NaCl, pH 7.4, 3 mM EDTA, 0.05% Tween 20) on a Biacore T200 unit. For ligand immobilisation Series S sensor chips CM5 and covalent amine coupling by using an Amine Coupling Kit were employed (all Cytiva, Freiburg, Germany). In initial experiments, either recombinant ReoM (20 μg/ml, diluted in 10 mM acetate buffer, pH 4.5) or recombinant MurA wt (20 μg/ml, diluted in 10 mM acetate buffer, pH 4.5) were immobilized on the surface of a sensor chip at high surface densities by injecting the diluted proteins over EDC/NHS-activated CM5 chips for 7 minutes at a flow rate of 10 μl per minute before blocking with 1.0 M ethanolamine-HCl pH 8.5. As a negative control, bovine serum albumin was coupled on the respective reference flow cells. Subsequently, in an initial binding experiment, either a 1:3 dilution series starting at 1 μM (46 μg/ml) MurA wt was injected over immobilized ReoM (3032 RU final response) and BSA (7715 RU) for 60 s at a flow rate of 30 μl/min followed by 300 s injections of HBS-EP+ for 300 s or, by switching orientation between immobilized ligand and analyte in solution, a 1:3 dilution series starting at 10 μM ReoM (210 μg/ml) was injected over immobilized MurA wt (5359 RU) and BSA (7520 RU) in a similar fashion. Optimum regeneration conditions to remove bound ReoM from immobilized MurA were determined by a regeneration scouting. Here, 10 mM glycin at pH 1.5 injected for 120 s at a flow rate of 10 μl/min completely removed ReoM as analyte while retaining the binding activity of immobilized MurA.

For kinetic binding analysis, the immobilisation levels of BSA on the control flow cell 1 (265 RU) and MurA N197D (flow cell 2, 512 RU), MurA S262L (flow cell 3, 754 RU) and MurA wt (flow cell 4, 525 RU) were lowered to a target immobilisation level of 500 resonance units (RU) to avoid mass transport limitation and avidity effects for binding of bivalent ReoM. ReoM was injected as analyte in solution for 120 s to monitor binding association followed by 600 s injection of HBS-EP+ to monitor complex dissociation. The highest concentration of ReoM was 280 μg/ml (13.3 μM) followed by six 1:3 dilutions with duplicate measurements of 4.3 μM. Regeneration was performed as determined in the regeneration scouting, all measurements were performed in two independent experiments. Double referenced binding curves [70] were fit to a 1:1: Langmuir interaction model using the Biacore Evaluation software (Cytiva, version 3.2) to determine association rate constants *k*_a_, dissociation rate constants *k*_d_ and the equilibrium dissociation constant *K*_D_.

### Lysis assays

Lysis assays were performed as described previously [71] with minor modifications. *L*. *monocytogenes* strains were grown in BHI broth at 37°C until an optical density of around OD_600_ ~1.0. Cells were collected by centrifugation (6000 x g, 5 min, 4°C) and the cell pellet was resuspended in 50 mM Tris/HCl pH8.0 to an optical density of OD_600_ = 1.0. Lysozyme was added to a final concentration of 2.5 μg /ml (where indicated) and the cells were shaken at 37°C. Lysis was followed by measuring optical density every 15 min in a spectrophotometer.

### Macrophage infection assay

Experiments to measure intracellular growth of *L*. *monocytogenes* strains inside J774 mouse macrophages were essentially carried out as described earlier [72,73].

### Microscopy

Cell membranes were stained through addition of 1 μl of nile red solution (100 μg ml^-1^ in DMSO) to 100 μl of exponentially growing bacteria. Images were taken with a Nikon Eclipse Ti microscope coupled to a Nikon DS-MBWc CCD camera and processed using the NIS elements AR software package (Nikon). Cell widths were determined using the tools for distance measurements provided by NIS elements AR.

For PG staining, strains from overnight cultures were diluted 1:50 in 500 μl BHI broth containing 0.1 mM 3-[[(7-Hydroxy-2-oxo-2H-1-benzopyran-3-yl)carbonyl]amino]-D-alanine hydrochloride (HADA) and grown overnight at 37°C. Cells were washed two times with 500 μl BHI broth and subjected to fluorescence microcopy. ImageJ was used to determine the fluorescence intensity maxima at the cell poles by densitometry. All values were corrected for background before further analysis.

Ultrathin section transmission electron microscopy was performed essentially as described earlier using a Tecnai 12 transmission electron microscope (Thermo Fisher/ FEI) operated at 120 kV [38].

## Supporting information

S1 Fig Suppression of the Δ*gpsB* phenotype by deletion of *prpC*. (A) Growth of *L*. *monocytogenes* strains EGD-e (wt), LMJR19 (Δ*gpsB*) and LMSW135 (Δ*gpsB* Δ*prpC*) in BHI broth at 42°C. Average values and standard deviations were calculated from an experiment performed in triplicate. (B) Western blots showing MurA and DivIVA levels (for control) in the same set of strains. MurA signals were quantified by densitometry and average values and standard deviations are shown (n = 3). Asterisks indicated statistically significant differences (*t*-test, *P*<0.01).
(TIF)

S2 Fig Effect of the N197D and S262L mutations on MurA activity. (A) Effect of the *murA N197D* and *S262L* mutations on growth of *L*. *monocytogenes*. Strains EGD-e (wt), LMJR123 (i*murA*), LMSW140 (i*murA N197D*) and LMSW141 (i*murA S262L*) were grown in BHI broth ± 1 mM IPTG at 37°C. IPTG-dependent strains had to be pre-depleted during a growth passage in the absence of IPTG to develop fully visible IPTG-dependence. Average values and standard deviations from an experiment performed in triplicate are shown. (B) Effect of the N197D and S262L mutations on fosfomycin susceptibility. The same strains as above were tested in a disc diffusion assay using filter discs soaked with fosfomycin on BHI agar plates not containing IPTG. The experiment was repeated three times and average values and standard deviations are shown. (C) Purification of MurA-Strep and its N197D and S262L variants. Proteins were purified to near homogeneity and aliquots were separated using a standard SDS polyacrylamide gel. (D) Enzymatic activity of MurA-Strep, MurAN197D-Strep and MurAS262L-Strep. Average values and standard deviations calculated from three repetitions are shown.
(TIF)

S3 Fig Effect of the *murA* mutations on suppression of the Δ*gpsB* phenotype. (A) Growth of *L*. *monocytogenes* strains EGD-e (wt) and LMJR19 (Δ*gpsB*) in BHI broth containing 1 mM IPTG at 42°C. (B-D) IPTG-dependent growth of *L*. *monocytogenes* strains LMJR117 (Δ*gpsB attB*::P_*help*_*-murA*, B), LMS307 (Δ*gpsB attB*::P_*help*_*-murA N197D*, C) and LMS306 (Δ*gpsB attB*::P_*help*_*-murA S262L*, D) in BHI broth supplemented with different IPTG concentrations at 42°C. One representative experiment out of three independent repetitions is shown.
(TIF)

S4 Fig MurA levels in strains used for pull-down. Western blot showing the levels of MurA in *L*. *monocytogenes* strains LMPR1 (labelled “wt”), LMPR13 (“S262L”) and LMPR14 (“N197D”) prior to formaldehyde treatment and pull-down analysis shown in Fig 3B.
(TIF)

S5 Fig *In vitro* interaction of ReoM with MurA, MurA N197D and MurA S262L. Double referenced sensorgrams (red) and results of fitting a 1:1 Langmuir binding model (black) to the interaction between a 1:3 dilution series of ReoM (starting at 13.3 μM) binding to immobilized MurA wt (A), MurA N197D (B), and MurA S262L (C) by surface plasmon resonance.
(TIF)

S6 Fig Thicker peptidoglycan in *murA* escape mutants. (A) Electron micrographs showing longitudinal sections of ultrathin-sectioned cells of *L*. *monocytogenes* strains EGD-e (wt), LMJR138 (Δ*clpC*), LMSW156 (*murA N197D*) and LMSW155 (*murA S262L*). Strains were grown in BHI broth at 42°C to early stationary phase (OD_600_ = 1.5). Strain LMJR116 (wt+*murA*), which contains a second IPTG-inducible copy of *murA*, was included as control. Scale bar is 500 nm. (B-C) Boxplots showing PG thickness at the cell poles (B) and the lateral wall (C). For determination of polar PG thickness, 25–29 longitudinally cut cells per strain were randomly selected and PG thickness was measured at three positions per pole, resulting in 96–166 measurements per strain. For determination of lateral PG thickness, 27–29 longitudinally cut cells per strain were selected and 10 measurements per cell were performed. Samples were blinded and mixed prior to the analysis. Asterisks mark statistical significance compared to wild type as the reference (*P*<0.01, *t*-test with Bonferroni-Holm correction). Median values are also shown.
(TIF)

S7 Fig Sensitivity of *murA* escape mutants against salt and lysozyme. (A) Growth of *L*. *monocytogenes* strains EGD-e (wt), LMJR138 (Δ*clpC*), LMSW155 (*murA S262L*) and LMSW156 (*murA N197D*) in BHI broth containing 5% (w/v) NaCl at 37°C. The experiment was performed in triplicate and average values and standard deviations are shown. (B) Lysis of the same set of strains in the presence of lysozyme. The experiment was performed three times, average values and standard deviations are shown.
(TIF)

S8 Fig Suppression of the *prkA* phenotype by *reoM*, *reoY* and *murZ* deletions. Minimal inhibitory ceftriaxone concentrations of *L*. *monocytogenes* strains EGD-e (wt), LMSW30 (Δ*reoM*), LMSW32 (Δ*reoY*), LMJR104 (Δ*murZ*), LMSW84 (i*prkA*), LMSW146 (Δ*prkA* Δ*reoM*), LMSW144 (Δ*prkA* Δ*reoY*) and LMSW145 (Δ*prkA* Δ*murZ*) are shown. Values represent average values from three repetitions. Asterisks mark statistical significance (*P*<0.05, *t*-test with Bonferroni-Holm correction).
(TIF)

S9 Fig Recreation of the *murA N197D* mutation confirms its role in ceftriaxone resistance and suppression of the *prkA* and *gpsB* phenotypes. (A) Ceftriaxone resistance of *L*. *monocytogenes strains* EGD-e (wt), LMSW156 (*murA N197D*, *gpsB* repaired suppressor strain) and LMS308 (recreated *murA N197D* mutant). The experiment was repeated three times and average values and standard deviations are shown. Asterisks mark statistical significance (*P*<0.01, *t*-test with Bonferroni-Holm correction). (B) Deletion of *prkA* in the recreated *murA N197D* mutant results in a viable strain. Growth of strains EGD-e, LMSW84 (i*prkA*), LMS308 (recreated *murA N197D* mutant) and LMS309 (*murA N197D* Δ*prkA*, obtained from LMS308 through *prkA* deletion) in BHI broth at 37°C. Average values and standard deviations were calculated from technical parallels (n = 5). (C) Suppression of the Δ*gpsB* growth defect at 42°C by the recreated *murA N197D* mutation. Growth of strains EGD-e, LMJR19 (Δ*gpsB*), LMS308 (recreated *murA N197D* mutant) and LMS310 (*murA N197D* Δ*gpsB*, obtained from LMS308 through *gpsB* deletion) in BHI broth at 42°C. Average values and standard deviations were calculated from technical parallels (n = 5).
(TIF)

S10 Fig Conservation of the PrkA signaling cascade in selected Gram-positive bacteria. Components of the PrkA signaling route in *L*. *monocytogenes* EGD-e (*Lmo*) and their homologues in *B*. *subtilis* 168 (*Bsu*), *E*. *faecalis* V583 (*Efa*), *S*. *aureus* N315 (*Sau*) and *S*. *pneumoniae* R6 (*Spn*). Locus numbers are given below the protein names and protein sequence homologies are shown as e-values (in brackets).
(TIF)
