# Supplementary figures and images for: MurA escape mutations uncouple peptidoglycan biosynthesis from PrkA signaling
Source: PLoS Pathog. 2022 Mar 16;18(3):e1010406. doi: 10.1371/journal.ppat.1010406 (PMC8959180; doi:10.1371/journal.ppat.1010406)

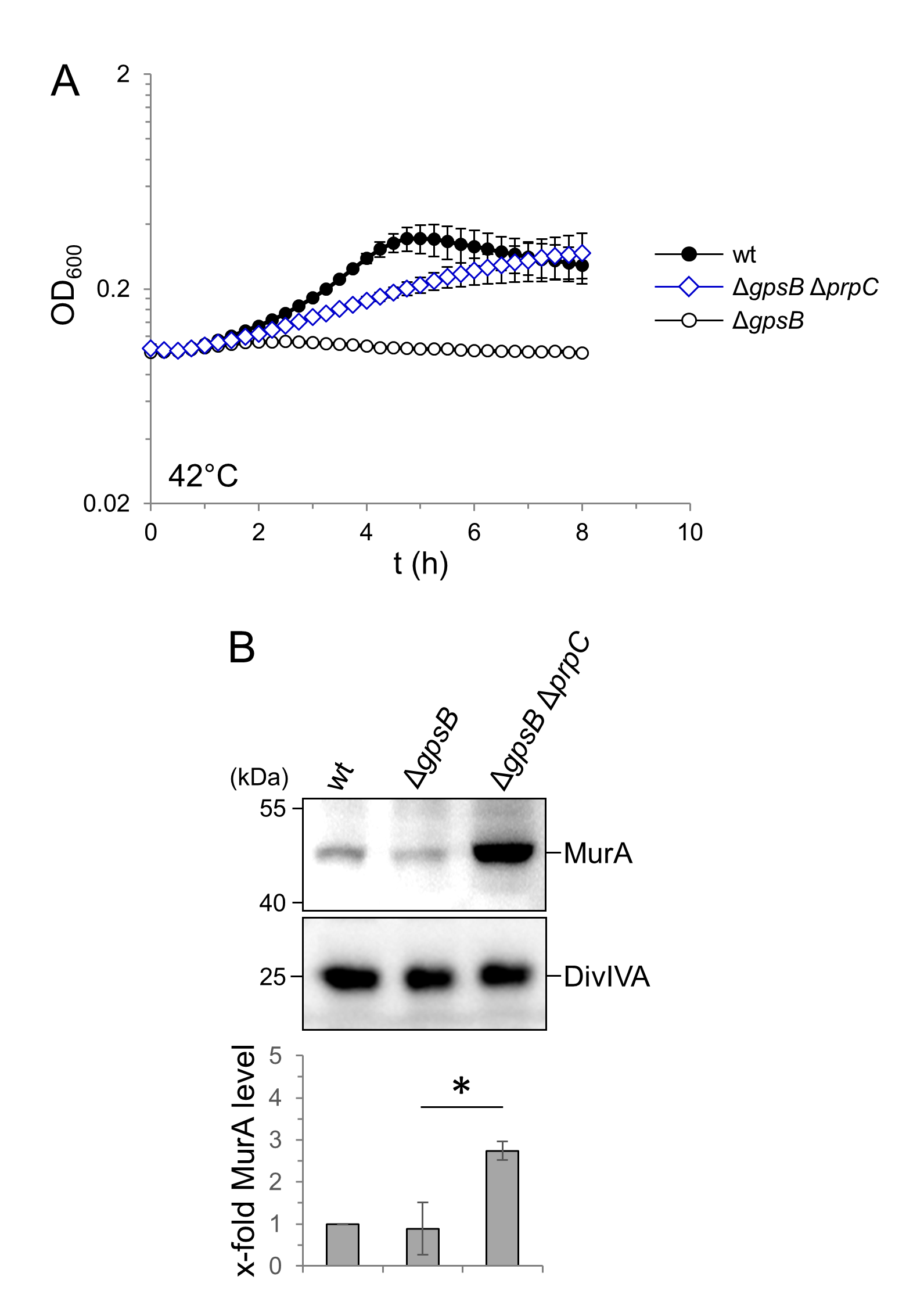

Supplement: S1 Fig — (A) Growth of L. monocytogenes strains EGD-e (wt), LMJR19 (ΔgpsB) and LMSW135 (ΔgpsB ΔprpC) in BHI broth at 42°C. Average values and standard deviations were calculated from an experiment performed in triplicate. (B) Western blots showing MurA and DivIVA levels (for control) in the same set of strains. MurA signals were quantified by densitometry and average values and standard deviations are shown (n = 3). Asterisks indicated statistically significant differences (t-test, P<0.01). (TIF) [file ppat.1010406.s001.TIF]

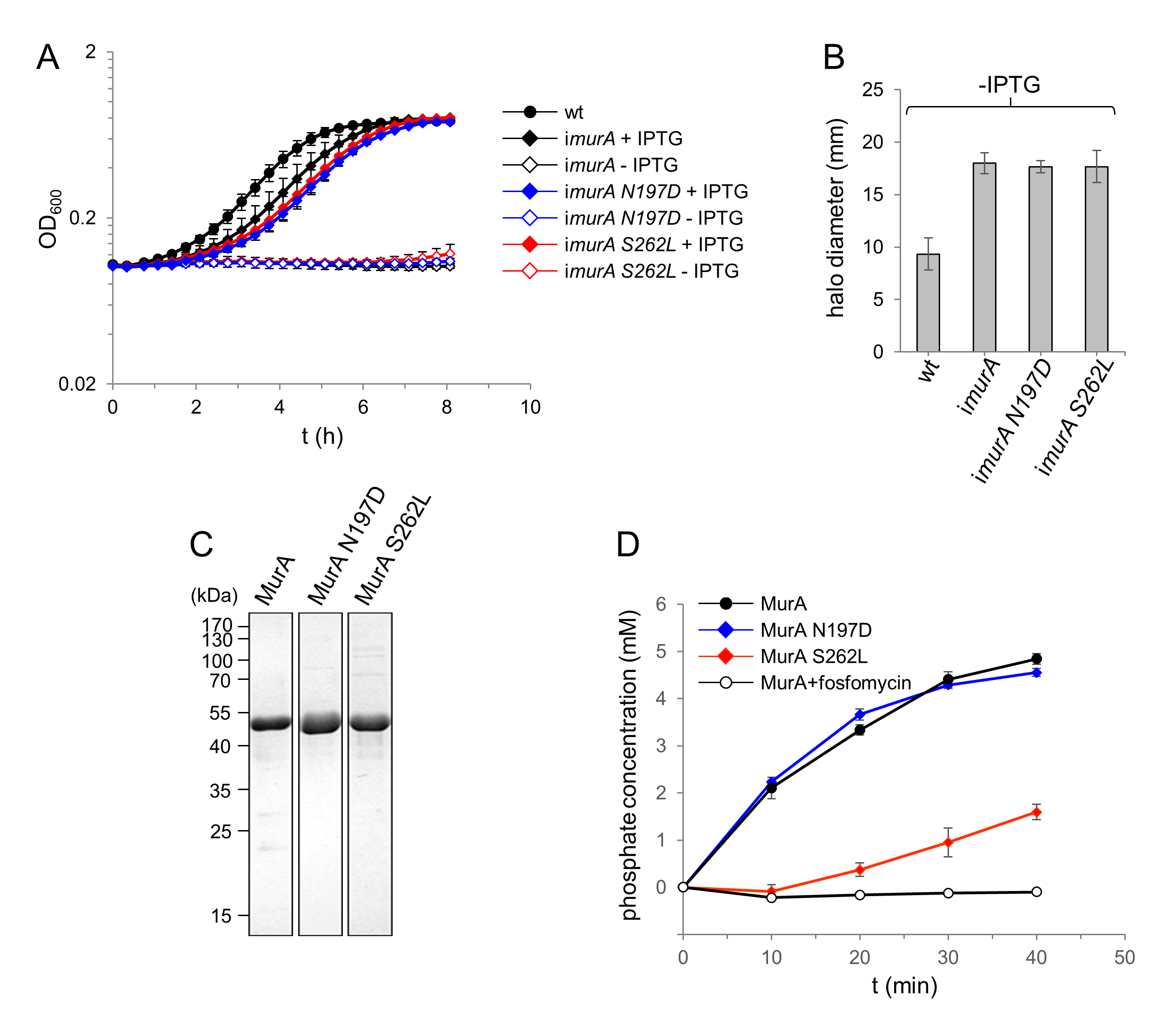

Supplement: S2 Fig — (A) Effect of the murA N197D and S262L mutations on growth of L. monocytogenes. Strains EGD-e (wt), LMJR123 (imurA), LMSW140 (imurA N197D) and LMSW141 (imurA S262L) were grown in BHI broth ± 1 mM IPTG at 37°C. IPTG-dependent strains had to be pre-depleted during a growth passage in the absence of IPTG to develop fully visible IPTG-dependence. Average values and standard deviations from an experiment performed in triplicate are shown. (B) Effect of the N197D and S262L mutations on fosfomycin susceptibility. The same strains as above were tested in a disc diffusion assay using filter discs soaked with fosfomycin on BHI agar plates not containing IPTG. The experiment was repeated three times and average values and standard deviations are shown. (C) Purification of MurA-Strep and its N197D and S262L variants. Proteins were purified to near homogeneity and aliquots were separated using a standard SDS polyacrylamide gel. (D) Enzymatic activity of MurA-Strep, MurAN197D-Strep and MurAS262L-Strep. Average values and standard deviations calculated from three repetitions are shown. (TIF) [file ppat.1010406.s002.TIF]

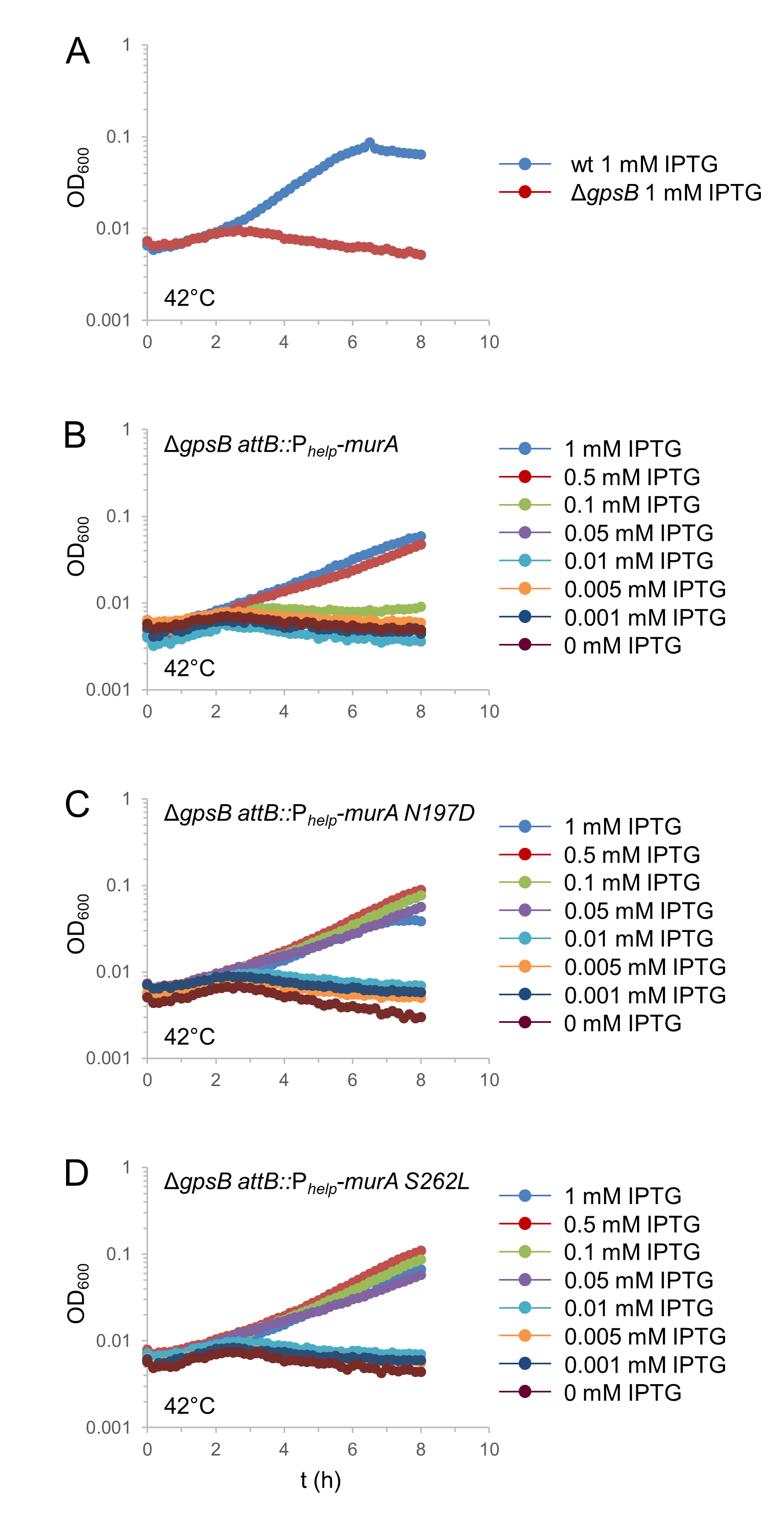

Supplement: S3 Fig — (A) Growth of L. monocytogenes strains EGD-e (wt) and LMJR19 (ΔgpsB) in BHI broth containing 1 mM IPTG at 42°C. (B-D) IPTG-dependent growth of L. monocytogenes strains LMJR117 (ΔgpsB attB::Phelp-murA, B), LMS307 (ΔgpsB attB::Phelp-murA N197D, C) and LMS306 (ΔgpsB attB::Phelp-murA S262L, D) in BHI broth supplemented with different IPTG concentrations at 42°C. One representative experiment out of three independent repetitions is shown. (TIF) [file ppat.1010406.s003.TIF]

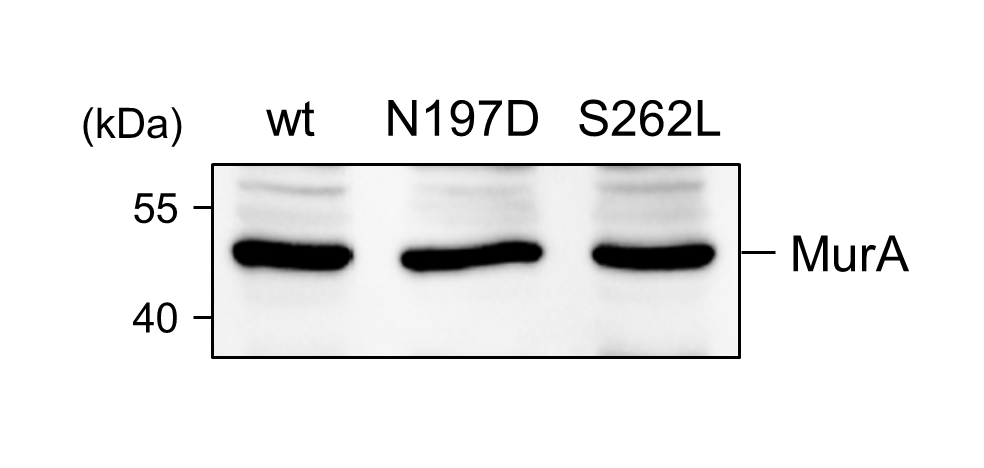

Supplement: S4 Fig — Western blot showing the levels of MurA in L. monocytogenes strains LMPR1 (labelled “wt”), LMPR13 (“S262L”) and LMPR14 (“N197D”) prior to formaldehyde treatment and pull-down analysis shown in Fig 3B. (TIF) [file ppat.1010406.s004.TIF]

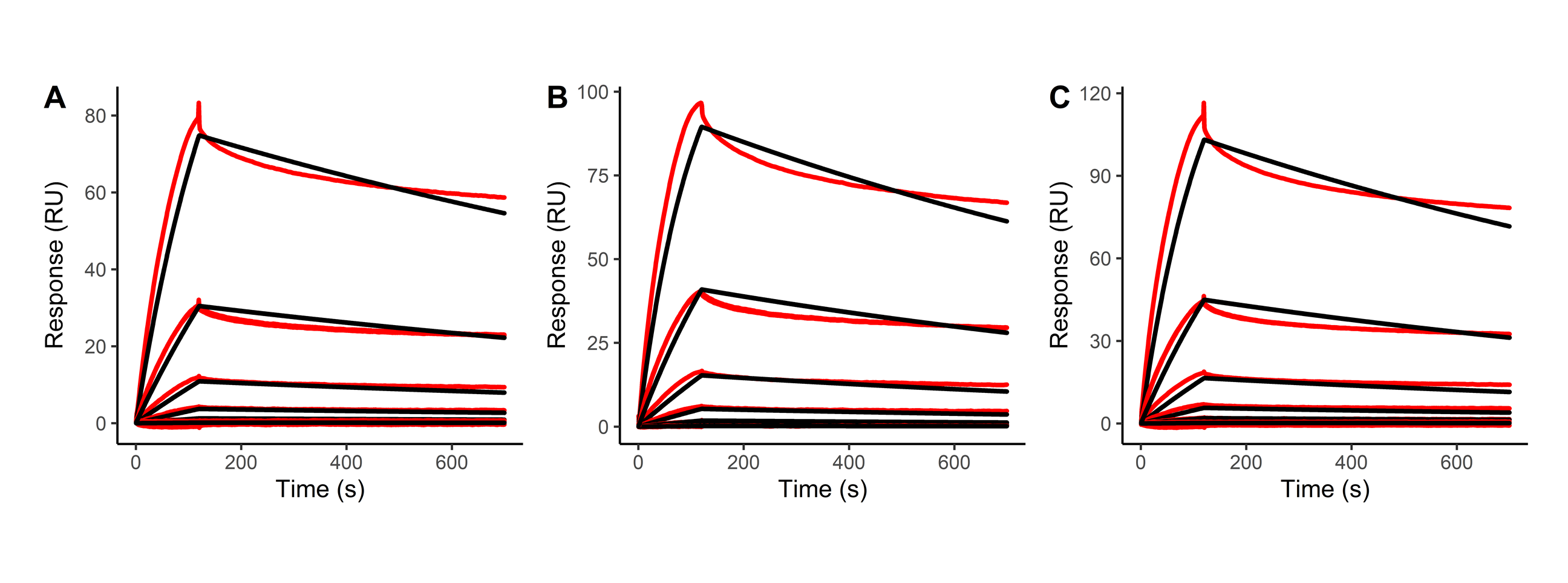

Supplement: S5 Fig — Double referenced sensorgrams (red) and results of fitting a 1:1 Langmuir binding model (black) to the interaction between a 1:3 dilution series of ReoM (starting at 13.3 μM) binding to immobilized MurA wt (A), MurA N197D (B), and MurA S262L (C) by surface plasmon resonance. (TIF) [file ppat.1010406.s005.TIF]

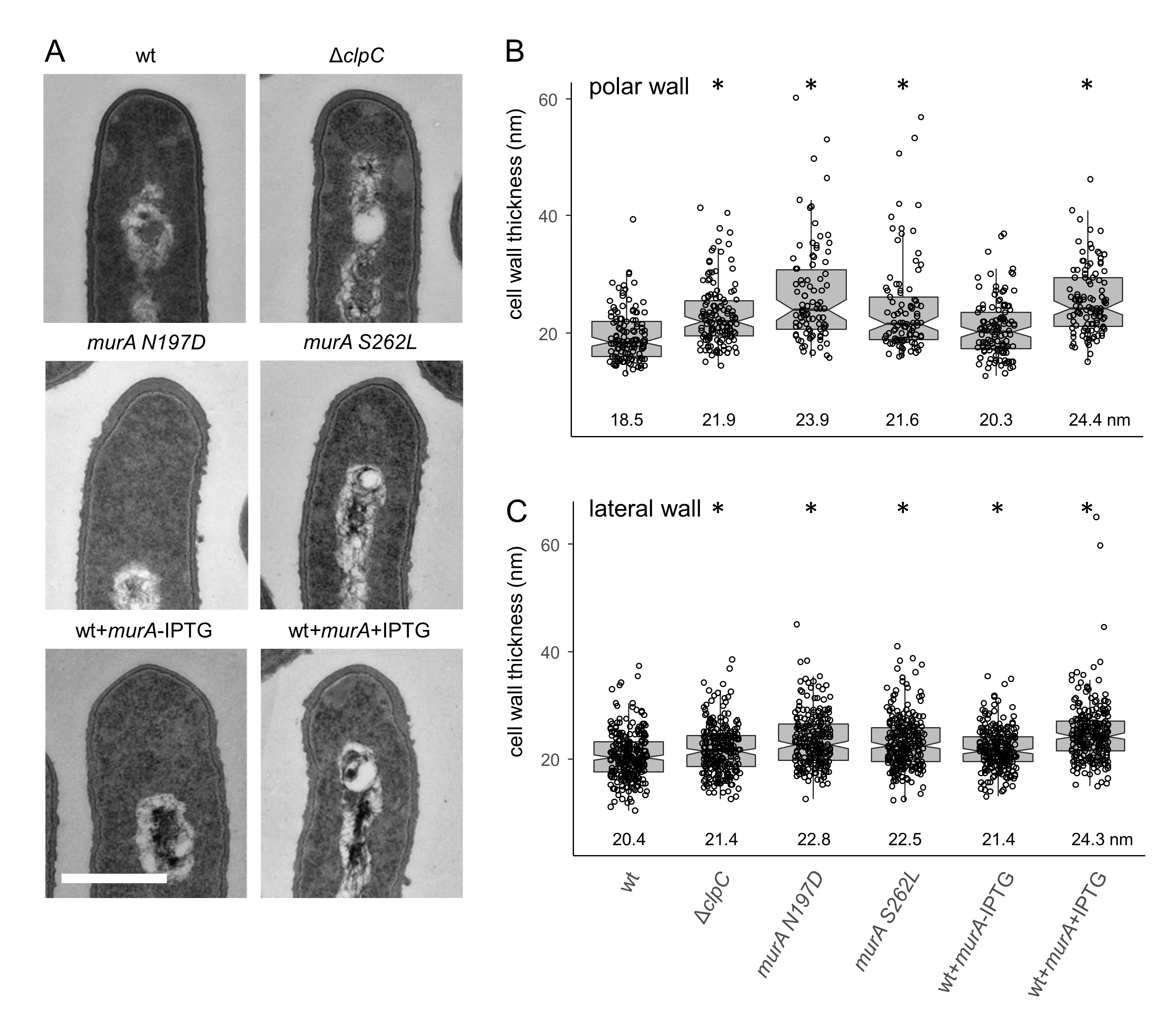

Supplement: S6 Fig — (A) Electron micrographs showing longitudinal sections of ultrathin-sectioned cells of L. monocytogenes strains EGD-e (wt), LMJR138 (ΔclpC), LMSW156 (murA N197D) and LMSW155 (murA S262L). Strains were grown in BHI broth at 42°C to early stationary phase (OD600 = 1.5). Strain LMJR116 (wt+murA), which contains a second IPTG-inducible copy of murA, was included as control. Scale bar is 500 nm. (B-C) Boxplots showing PG thickness at the cell poles (B) and the lateral wall (C). For determination of polar PG thickness, 25–29 longitudinally cut cells per strain were randomly selected and PG thickness was measured at three positions per pole, resulting in 96–166 measurements per strain. For determination of lateral PG thickness, 27–29 longitudinally cut cells per strain were selected and 10 measurements per cell were performed. Samples were blinded and mixed prior to the analysis. Asterisks mark statistical significance compared to wild type as the reference (P<0.01, t-test with Bonferroni-Holm correction). Median values are also shown. (TIF) [file ppat.1010406.s006.TIF]

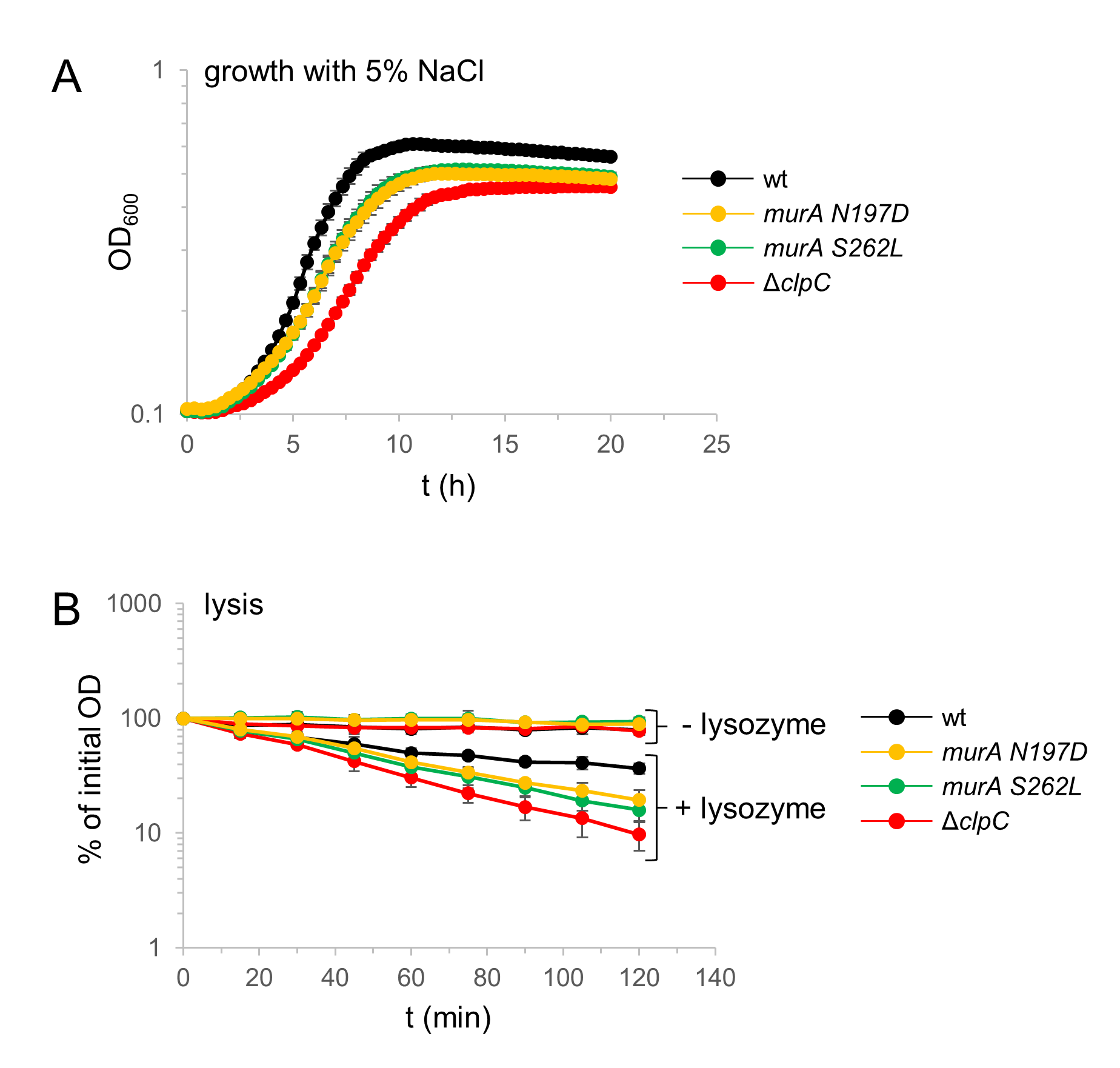

Supplement: S7 Fig — (A) Growth of L. monocytogenes strains EGD-e (wt), LMJR138 (ΔclpC), LMSW155 (murA S262L) and LMSW156 (murA N197D) in BHI broth containing 5% (w/v) NaCl at 37°C. The experiment was performed in triplicate and average values and standard deviations are shown. (B) Lysis of the same set of strains in the presence of lysozyme. The experiment was performed three times, average values and standard deviations are shown. (TIF) [file ppat.1010406.s007.TIF]

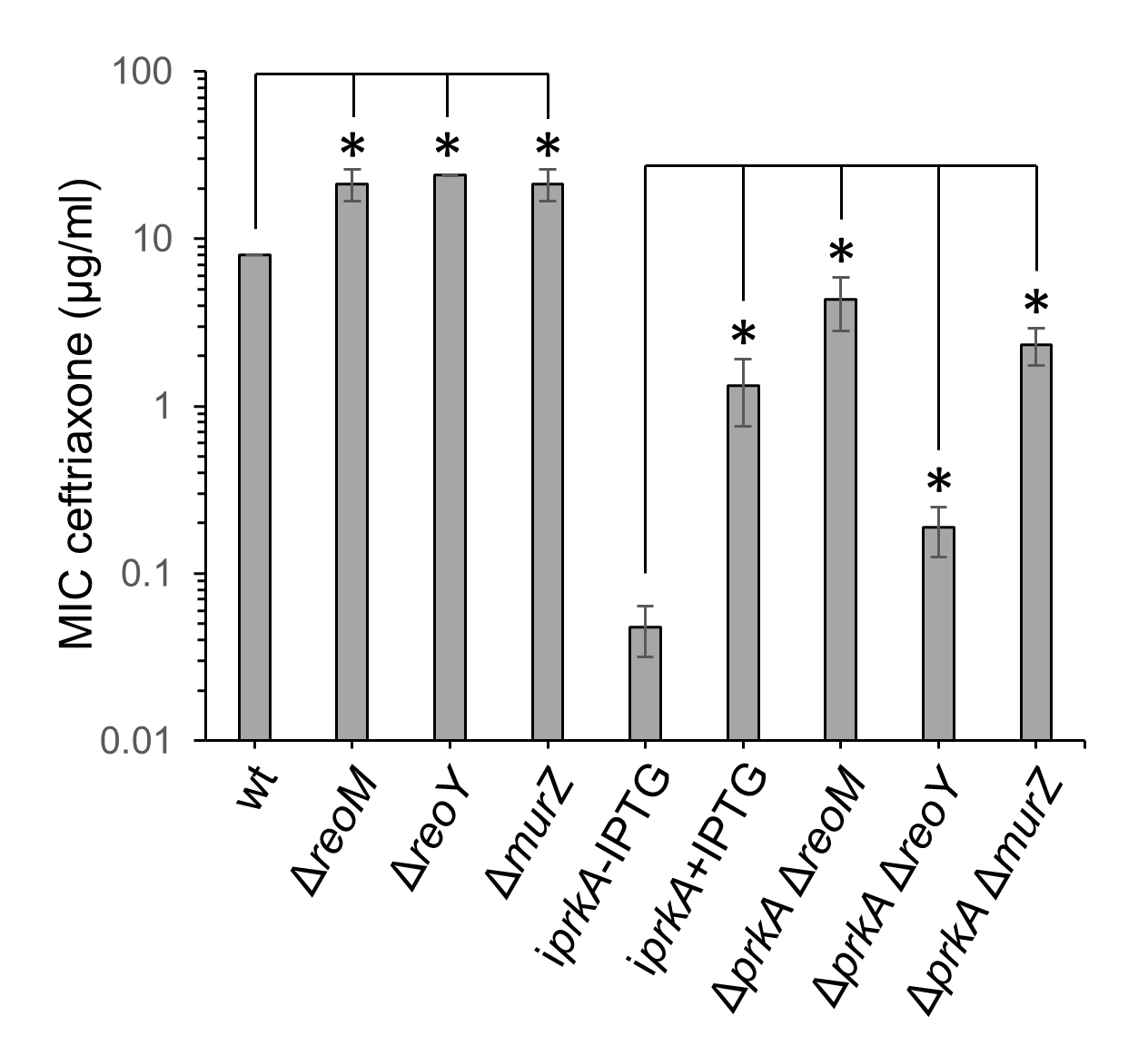

Supplement: S8 Fig — Minimal inhibitory ceftriaxone concentrations of L. monocytogenes strains EGD-e (wt), LMSW30 (ΔreoM), LMSW32 (ΔreoY), LMJR104 (ΔmurZ), LMSW84 (iprkA), LMSW146 (ΔprkA ΔreoM), LMSW144 (ΔprkA ΔreoY) and LMSW145 (ΔprkA ΔmurZ) are shown. Values represent average values from three repetitions. Asterisks mark statistical significance (P<0.05, t-test with Bonferroni-Holm correction). (TIF) [file ppat.1010406.s008.TIF]

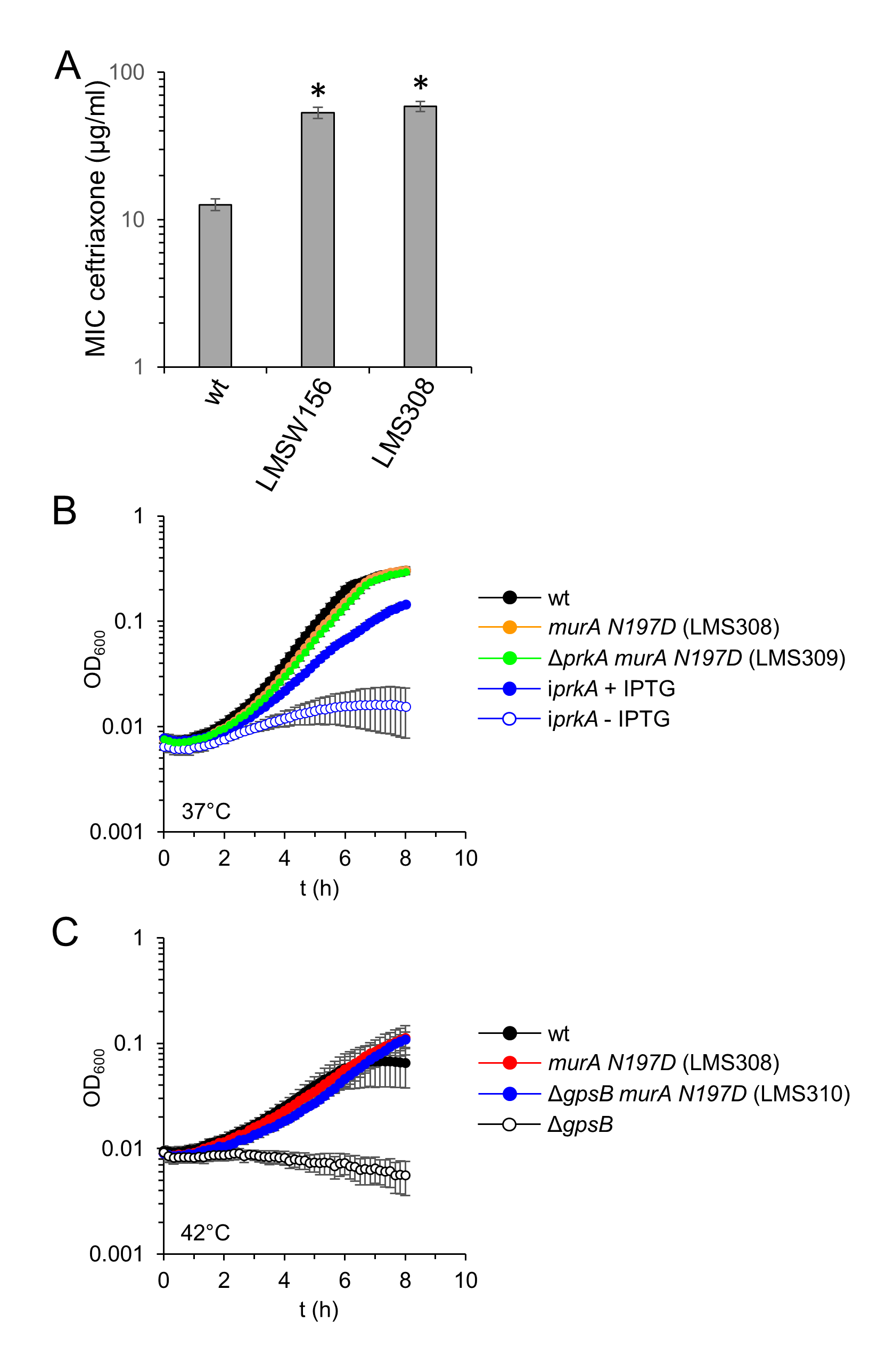

Supplement: S9 Fig — (A) Ceftriaxone resistance of L. monocytogenes strains EGD-e (wt), LMSW156 (murA N197D, gpsB repaired suppressor strain) and LMS308 (recreated murA N197D mutant). The experiment was repeated three times and average values and standard deviations are shown. Asterisks mark statistical significance (P<0.01, t-test with Bonferroni-Holm correction). (B) Deletion of prkA in the recreated murA N197D mutant results in a viable strain. Growth of strains EGD-e, LMSW84 (iprkA), LMS308 (recreated murA N197D mutant) and LMS309 (murA N197D ΔprkA, obtained from LMS308 through prkA deletion) in BHI broth at 37°C. Average values and standard deviations were calculated from technical parallels (n = 5). (C) Suppression of the ΔgpsB growth defect at 42°C by the recreated murA N197D mutation. Growth of strains EGD-e, LMJR19 (ΔgpsB), LMS308 (recreated murA N197D mutant) and LMS310 (murA N197D ΔgpsB, obtained from LMS308 through gpsB deletion) in BHI broth at 42°C. Average values and standard deviations were calculated from technical parallels (n = 5). (TIF) [file ppat.1010406.s009.TIF]

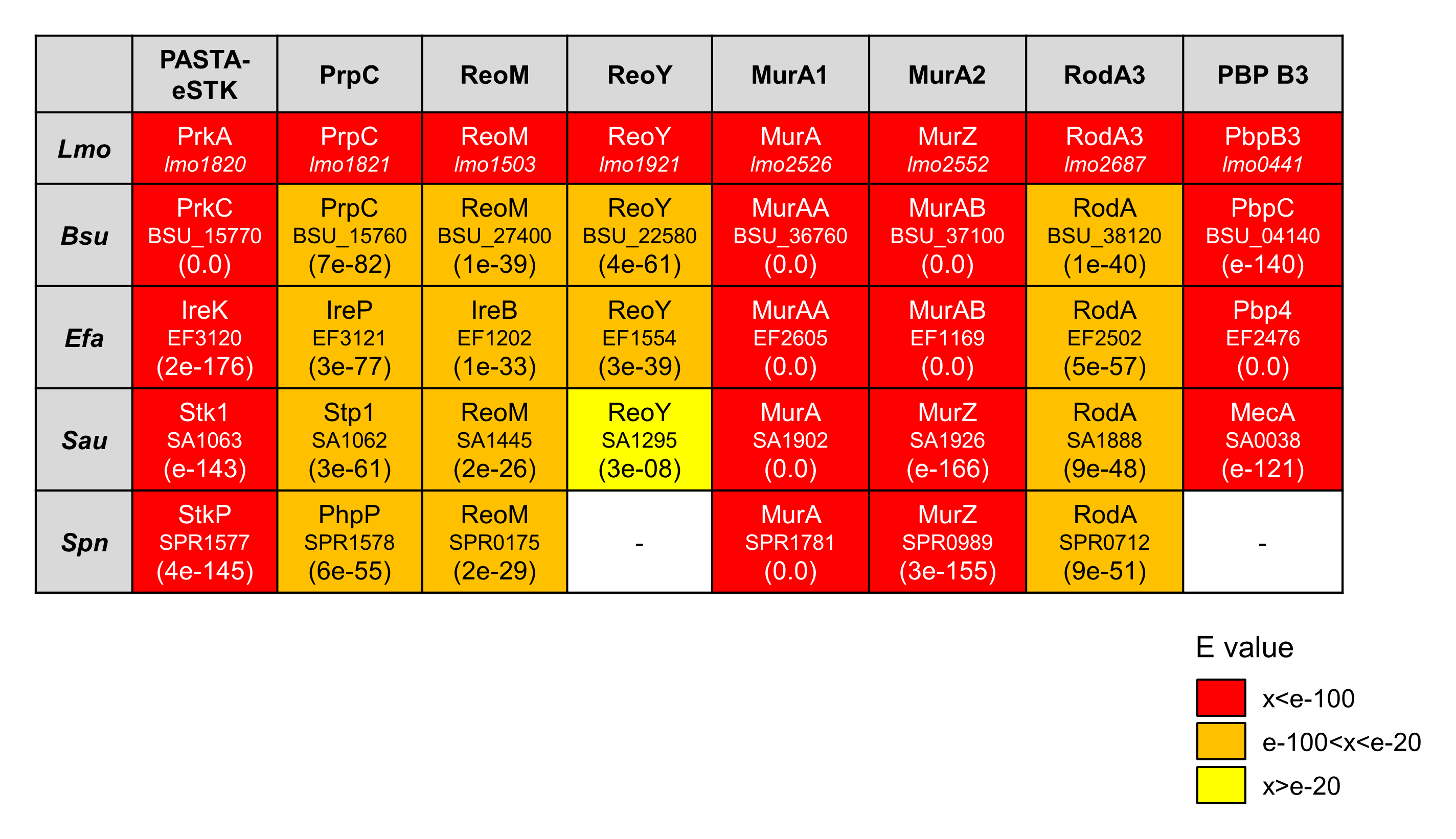

Supplement: S10 Fig — Components of the PrkA signaling route in L. monocytogenes EGD-e (Lmo) and their homologues in B. subtilis 168 (Bsu), E. faecalis V583 (Efa), S. aureus N315 (Sau) and S. pneumoniae R6 (Spn). Locus numbers are given below the protein names and protein sequence homologies are shown as e-values (in brackets). (TIF) [file ppat.1010406.s010.TIF]
